# An open-source camera-based trifilar pendulum setup for measuring mass moment of inertia of small satellites

**DOI:** 10.1016/j.ohx.2026.e00821

**Published:** 2026-09-11

**Authors:** Bas Stijnen, Joseph Thompson, Ryan Paetzold, Eoghan Somers, David McKeown

**Affiliations:** University College Dublin School of Mechanical and Materials Engineering, University College Dublin, Belfield, Dublin 4, D04 V1W8, Ireland

**Keywords:** CubeSat, Mass moment of inertia, Trifilar pendulum, Optical tracking, Open-source hardware, Additive manufacturing

## Abstract

Given the growing use of CubeSats and other small satellites, direct measurement of mass properties such as the mass moment of inertia (MoI) has become increasingly important for attitude-control applications. Although CAD and finite-element models are widely used to estimate spacecraft mass properties, their accuracy depends on model fidelity and may omit practical assembly details. This paper presents an open-source, camera-based trifilar pendulum for measuring the MoI of CubeSat-scale hardware and other compact rigid bodies. The system comprises an additively manufactured platform, a camera, and custom analysis software, with a hardware cost of approximately €55. Depending on the required accuracy and budget, either a low-cost webcam or a GoPro camera may be used in combination with steel or Dyneema suspension lines. The accompanying software provides optical tracking, automated MoI calculation, centre-of-mass offset compensation, uncertainty estimation, and a graphical user interface. Validation using calibration masses with known theoretical inertias and a representative CubeSat-like test article showed measurement errors below 5% for MoI values above $0.003\;\mathrm{kg}\;m^{2}$ and a 7.2% deviation from an independently generated CAD model. All hardware designs, software, and assembly documentation are openly available to support reproduction and further development

## Highlights

• Open-source trifilar pendulum for low-cost inertia measurements.
• Camera-based optical tracking eliminates contact measurement methods.
• Additively manufactured platform designed for small satellite testing.
• Validation performed using reference masses over a wide inertia range.
• Complete hardware, software, and assembly files are publicly available.

## Graphical abstract

**Figure:**
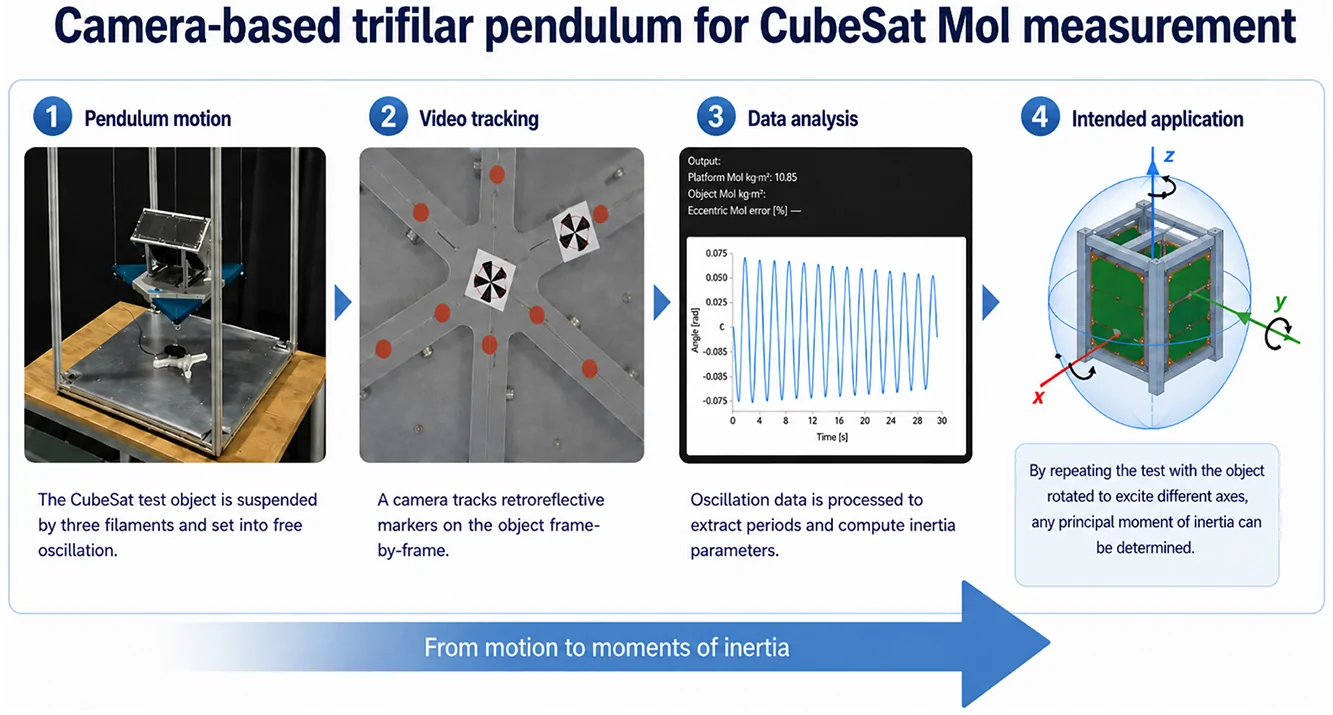

**Table:** 

**Specifications table**	

**Hardware name**	Trifilar Pendulum for MoI Measurements of Small Objects

**Subject area**	Engineering and Material Science

**Hardware type**	Measuring physical properties and in-lab sensors

**Closest commercial analog**	*Tecquipment - Lab equipment for studying harmonic motion in trifilar pendulums*[1]

**Open source license**	*CC-BY-4.0, MIT License, CERN-OHL*

**Cost of hardware**	€55 to €550

**Source file repository**	https://doi.org/10.17632/zww548rfbn.6

**OSHWA certification UID**	IE000005

## 1 Hardware in context

Accurate knowledge of the mass moment of inertia (MoI) is essential for the attitude analysis, control design, and verification of small satellites such as CubeSats [2] and PocketQubes [3]. Although MoI can be estimated from CAD or finite element models, the resulting values depend strongly on model fidelity and often omit practical details such as fasteners, harnessing, manufacturing tolerances, and late-stage hardware changes. For small spacecraft, where compact packaging and tightly constrained margins make the mass properties especially sensitive to such details, direct experimental measurement is therefore highly valuable. The trifilar pendulum is widely established as one of the most accessible and accurate methods for measuring the mass moment of inertia (MoI) [4], [5], [6], [7]. Trifilar pendulum systems represent only one class of inertia measurement techniques. Alternative approaches include tetrahedron-based measurement methods [8], multi-cable pendulum systems [7], five-wire torsion pendulums with optical sensing [9], and pendulum-based methods capable of simultaneously determining centre of gravity and inertia tensor properties [10]. Spacecraft-specific inertia measurement systems have also been reported for both large space payloads [11] and small satellites [12]. In a typical configuration, a platform is suspended by three equal-length wires and given a small angular displacement. The oscillation period is then used to determine the rotational inertia of the suspended system. Related systems have been reported previously, including the design described by Titchener [13]. In the present work, a homogeneous platform, with its centre of mass (CoM) aligned with its geometric centre (GC), is suspended from a structure or ceiling using three equal-length, pre-stretched wires, allowing it to oscillate freely. A small rotational displacement is applied to the platform, and the resulting oscillation period is analysed to determine the MoI. By tracking markers attached to the underside of the platform using a camera, the oscillation period can be measured precisely without introducing additional damping to the system. The MoI is then calculated using the standard trifilar pendulum relation described in Section 6. Optical tracking was selected as the primary measurement technique because it provides a non-contact method of determining the oscillation period without introducing additional mass, friction, or damping into the system. Alternative approaches, such as rotary encoders or inertial sensors mounted to the platform, require physical attachment to the oscillating body and may alter its dynamic properties. By using a camera-based system, measurements can be obtained without modifying the item under test, while maintaining a relatively low hardware cost. The optical tracking system employs N-fold fiducial markers developed by [14]. Camera-based fiducial markers are widely used for object identification and pose estimation applications because they enable robust tracking using a single camera system [15]. The N=4 and N=5 markers used in this work were selected based on the MarkerLocator framework proposed by [14]. Their differing rotational symmetry orders allow the two markers to be uniquely identified and distinguished during image processing while providing reliable rotational pose estimation. Compared with simpler geometric targets, N-fold markers offer improved robustness against rotational ambiguities and can be detected over a wide range of viewing angles and lighting conditions. The use of printed fiducial markers also avoids the need for specialised motion-capture equipment, contributing to the low-cost, portable, and reproducible nature of the proposed measurement system.

This system is suitable for measuring the combined MoI of both the platform and a test object, provided that the CoM of the object is aligned with that of the platform. The MoI of the test object can then be obtained by subtracting the platform MoI from the combined value. Given the interdependence between the platform and test object MoI, several design configurations are considered in this paper.

Commercial MoI measurement systems, such as those offered by Tecquipment® [1], are available but are often prohibitively expensive for early-stage CubeSat projects. In addition, while several studies focus on MoI measurements for larger systems such as boats, aircraft, engines, and cars [4], [7], relatively little literature addresses mass property measurements for small satellites, such as CubeSats and PocketQubes. This creates a gap between the need for experimentally validated mass properties and the availability of affordable, reproducible tools.

This work addresses that gap by presenting an open-source, camera-based trifilar pendulum system designed primarily for small satellites, while remaining suitable for other small rigid bodies. While a number of inertia measurement systems have been reported in the literature, including systems developed specifically for spacecraft and small-satellite applications [11], [12], many are either custom laboratory installations, proprietary commercial products, or lack sufficient documentation to enable straightforward reproduction. In contrast, the present work provides openly accessible design files, assembly instructions, software, and validation data, allowing the complete system to be reproduced and modified by other researchers. The contribution lies not in the trifilar principle itself, but in the combination of a portable test platform, low-cost optical tracking, additively manufactured components, and openly available analysis software and design files. This emphasis on accessibility and reproducibility is particularly relevant for university CubeSat programmes and small research groups operating under limited budgets. The following section describes the hardware architecture and the practical design choices made to support repeatable and accessible MoI measurements.

The primary objective of this work is to develop and validate a low-cost, open-source measurement system capable of determining the mass moment of inertia of CubeSat-class spacecraft using a trifilar pendulum. A secondary objective is to provide accompanying software and documentation that allow the system to be reproduced and operated without specialised metrology equipment. The main contribution of this paper is the presentation of a complete open-source hardware and software solution for experimental MoI determination, including the mechanical design, image-processing software, validation methodology, and performance evaluation using reference objects with known inertial properties. By making all design files, software, and documentation publicly available, the work aims to lower the barrier to accurate spacecraft mass property measurements for universities, research laboratories, and small satellite developers.

## 2 Hardware description

**Fig. 1:**
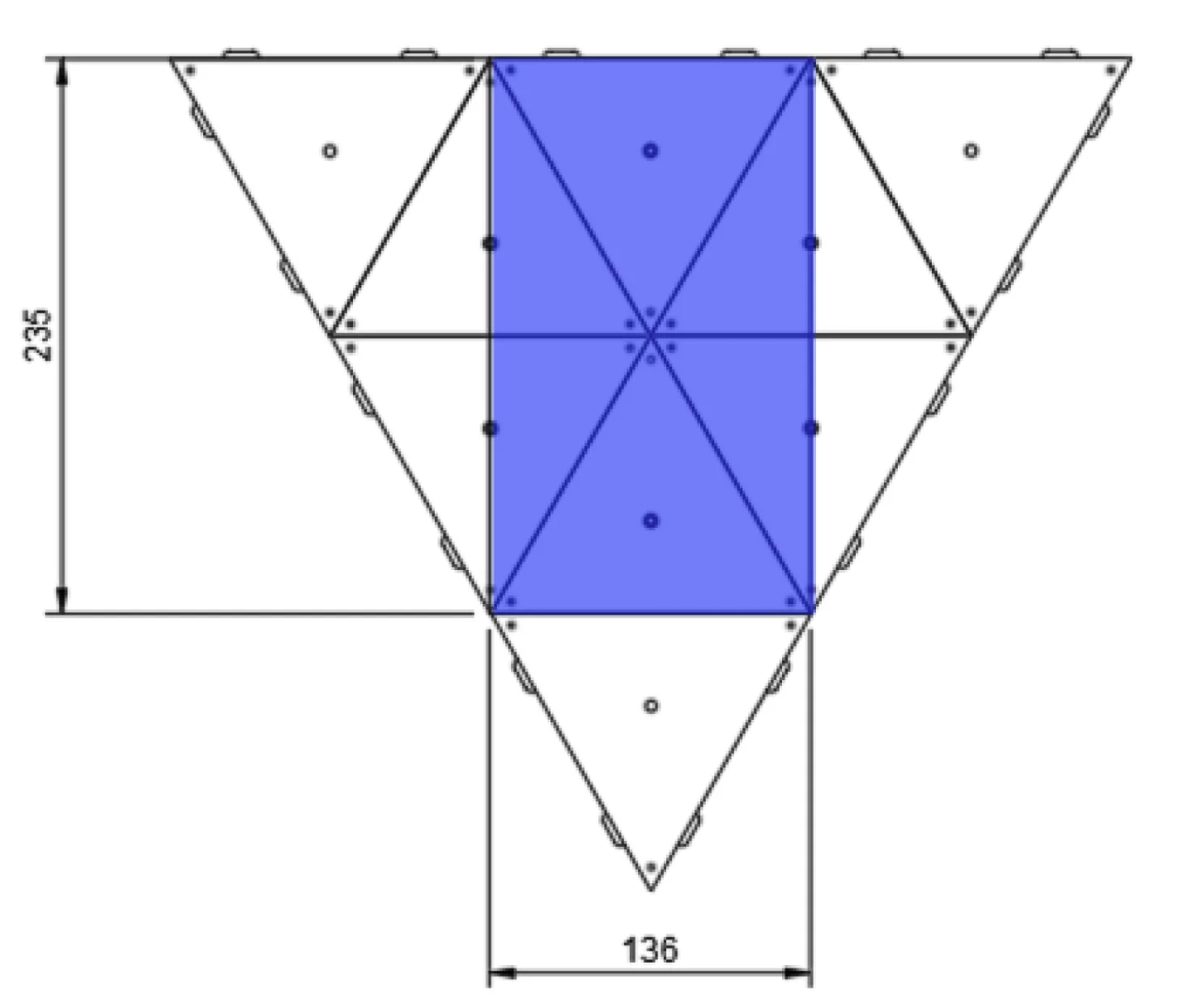
Useable measurement envelope of the trifilar platform. The shaded rectangle indicates the largest centred rectangular test article that can be accommodated while preserving alignment between the platform centre and the centre of the item under test.

The present configuration was designed and experimentally evaluated for compact test articles like CubeSats, with masses up to approximately (2kg). This value should be interpreted as a recommended operating range for the validated configuration rather than as a certified structural load limit. The suspension lines and fittings have load ratings above this value; however, for the additively manufactured platform the limiting factor is expected to be platform stiffness and local deformation around the bolted tile connections and suspension attachment points. Concentrated loads near the centre of the platform may introduce flexure and alter the effective suspension geometry, which would directly affect the measured oscillation period and hence the calculated MoI. Larger or more uniformly distributed test articles may therefore be feasible, but operation above (2kg) should be validated separately for the specific platform, print settings, and load distribution used. The useable platform area corresponds to an equilateral triangular footprint with a side length of approximately 407 mm, shown in Fig. 1. Test articles should remain within this footprint and should not extend beyond the suspension attachment points to avoid interference with the suspension lines during oscillation.

The measurement principle incorporates the use of a camera to track the angular motion of the suspended, additively manufactured platform using two n-fold visual markers [14] (see Fig. 3). The N=4 and N=5 markers were selected based on the MarkerLocator framework developed by [14]. The markers possess different rotational symmetry orders, allowing them to be uniquely identified and distinguished from one another during image processing. The use of markers with coprime symmetry orders improves robustness against rotational ambiguities while maintaining reliable pose estimation from a single camera. This enables accurate tracking of the platform orientation throughout the oscillation without requiring specialised motion-capture equipment. The accompanying software extracts the oscillation period from the recorded motion (as described in Section 6). By combining additive manufacturing with a user-friendly, portable visual data acquisition system, this project provides an accurate method for determining the MoI of objects within the size limitations of the platform. The key benefits of this test setup include:

**Fig. 2:**
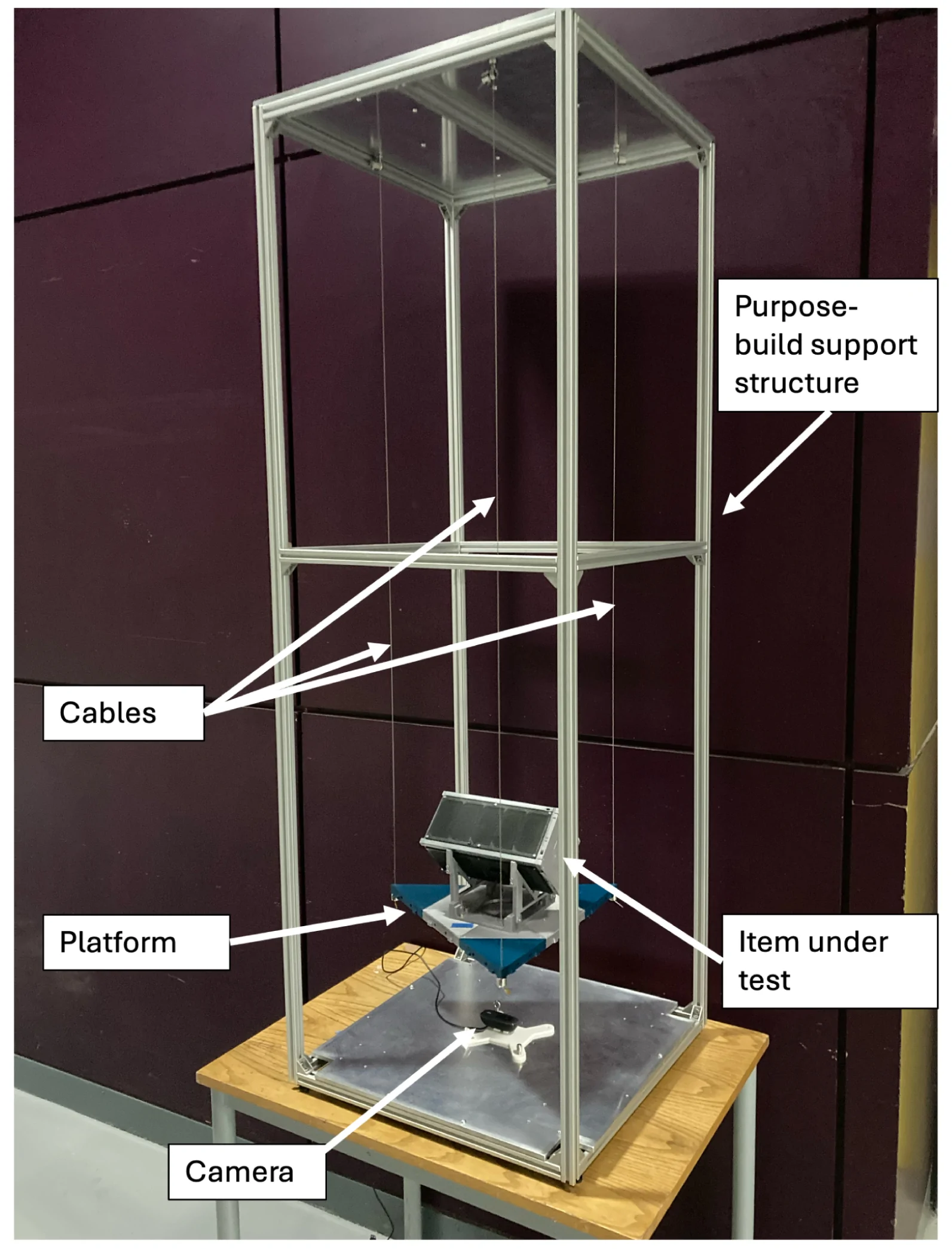
Trifilar pendulum including a purpose-built support structure.

• **Accurate MoI measurement:** Suitable for small objects such as CubeSats, PocketQubes, and RC aircraft, enabling improved understanding of their dynamic behaviour.
• **Educational value:** The simple, hands-on experimental setup can be used as a teaching tool to support understanding of mass properties.
• **Versatile video analysis:** The video tracking system can be adapted for use in other applications requiring motion tracking of rigid bodies.

The project consists of two main physical components: the trifilar platform, designed to hold the test object, and a support structure that stabilises the platform and mounts the camera. Although a portable support structure was developed to facilitate deployment in laboratory and cleanroom environments, including typical ISO 8 CubeSat assembly areas, the trifilar platform itself can also be suspended directly from existing ceiling-mounted attachment points. In such configurations the platform remains fully portable while avoiding potential influences introduced by the support frame. The support structure should therefore be considered an optional convenience feature rather than a fundamental requirement of the measurement system.

**Fig. 3:**
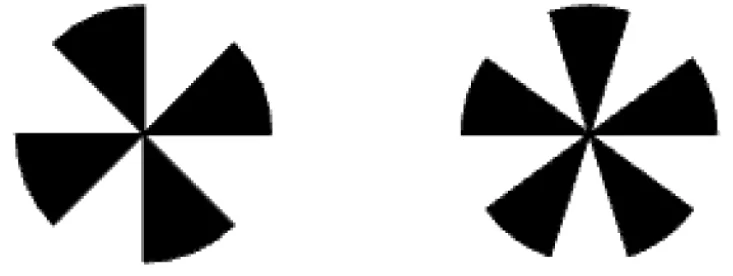
N-fold markers with N=4 (left) and N=5 (right).

## 3 Design files

All design files and accompanying documents are available via the Mendeley Data repository (DOI: https://doi.org/10.17632/zww548rfbn.6). The software required for the setup can be accessed on GitHub [16]. The design files are provided as either technical drawings or object files compatible with Blender™ [17], while supplementary documents are available in DOCX or XLSX formats. While this paper provides an overview, the complete set of platform and support structure design files, together with detailed assembly instructions and the bill of materials (BoM), are available in the online repository and provide more detail than can be presented here. A summary of these files is given in Table 1.

*Platform_tile* refers to a section of the platform, which is assembled from multiple triangular components designed for 3D printing using a fused filament fabrication (FFF) desktop printer. The minimum required build volume is 180 mm × 180 mm × 180 mm. *Aluminium top plate* is the design file for the top plate of the support structure, which accommodates the cable connections, while *Aluminium bottom plate* defines the bottom plate used to mount equipment such as a camera. *Markers N4 and N5* are n-fold markers used for motion tracking. These should be printed on a standard printer and attached to the underside of the platform using double-sided tape. Additional design files, such as *GoPro_mount* and *webcam_mount*, can be 3D printed and are intended for mounting a GoPro or webcam to the bottom plate of the structure. For visual reference or customisation, a fully assembled object file of the pendulum and support structure is available in the project database. This file can be opened using the open-source software Blender® . Finally, the last design file listed is the complete bill of materials (BoM), which provides a detailed list of all required components.

**Table 1:** Summary of design files.

Design filename	File type	Open source license	Location of the file
Platform_tile	Object file/STL/STEP	CERN-OHL	DOI: https://doi.org/10.17632/zww548rfbn.6 /Design files
Aluminium top plate technical drawing	PDF	CERN-OHL	DOI: https://doi.org/10.17632/zww548rfbn.6 /Design files
Aluminium bottom plate technical drawing	PDF	CERN-OHL	DOI: https://doi.org/10.17632/zww548rfbn.6 /Design files
Markers N4 and N5	DOCX	CC BY 4.0	DOI: https://doi.org/10.17632/zww548rfbn.6 /Design files
2GoPro_mount	Object file	CERN-OHL	DOI: https://doi.org/10.17632/zww548rfbn.6 /Design files
webcam_mount	Object file	CERN-OHL	DOI: https://doi.org/10.17632/zww548rfbn.6 /Design files
Trifilar Pendulum Assembly	Object file/STL/STEP	CERN-OHL	DOI: https://doi.org/10.17632/zww548rfbn.6 /Design files
Bill of Materials	XLSX	CC BY 4.0	DOI: https://doi.org/10.17632/zww548rfbn.6 /Design files
Software	PYTHON	MIT License	DOI: https://doi.org/10.17632/zww548rfbn.6 /Software

## 4 Bill of materials summary

The bill of materials (BoM) is divided into two sections: one for the pendulum and one for the supporting frame. Both are available in the online repository: https://doi.org/10.17632/zww548rfbn.6. The BoM for the pendulum is detailed in Table 2.

To perform an MoI measurement, the mass of both the test object and the platform must be known. Therefore, a weighing scale should be available, or the masses should be determined in advance. The bill of materials presented in Table 2 corresponds to the trifilar pendulum platform only. The optional support structure, camera, and host computer are listed separately in the complete repository BoM https://doi.org/10.17632/zww548rfbn.6. Depending on the selected configuration, the total system cost ranges from approximately 55 EUR for the pendulum platform alone to approximately 550 EUR for a complete setup including the support structure and camera. A standard webcam or action camera, together with a computer capable of running the analysis software, is required for operation but may already be available in many laboratory environments.

**Table 2:** Bill of materials.

Designator	Component	Number	Cost per unit - currency	Total cost - currency	Source of materials	Material type
Part # 1	Triangular platform tile	9	€1.49	€13.14	Farnell	Polymer (PLA)
Part # 2	Thermal threaded inserts M4	8	–	€17.79	Amazon	Metal
Part # 3	Steel wire 1260 mm lengths D 1mm 7 × 7	3	€0.53	€2.65	Tecni	Metal
Part # 4	Stop fitting	6	€2.86	€17.34	Tecni	Metal
Part # 5	Markers	2	€0.00^a^	€0.00	123ink.ie	Paper
Part # 6	M4 × 16 socket screws	8	€0.33	€2.66	Radionics	metal
part # 7	Tergo braid line Dyneema	1	€12.50	€12.50	fishing mega store	Dyneema®

a Material included in general office/laboratory supplies.

## 5 Build instructions

**Fig. 4:**
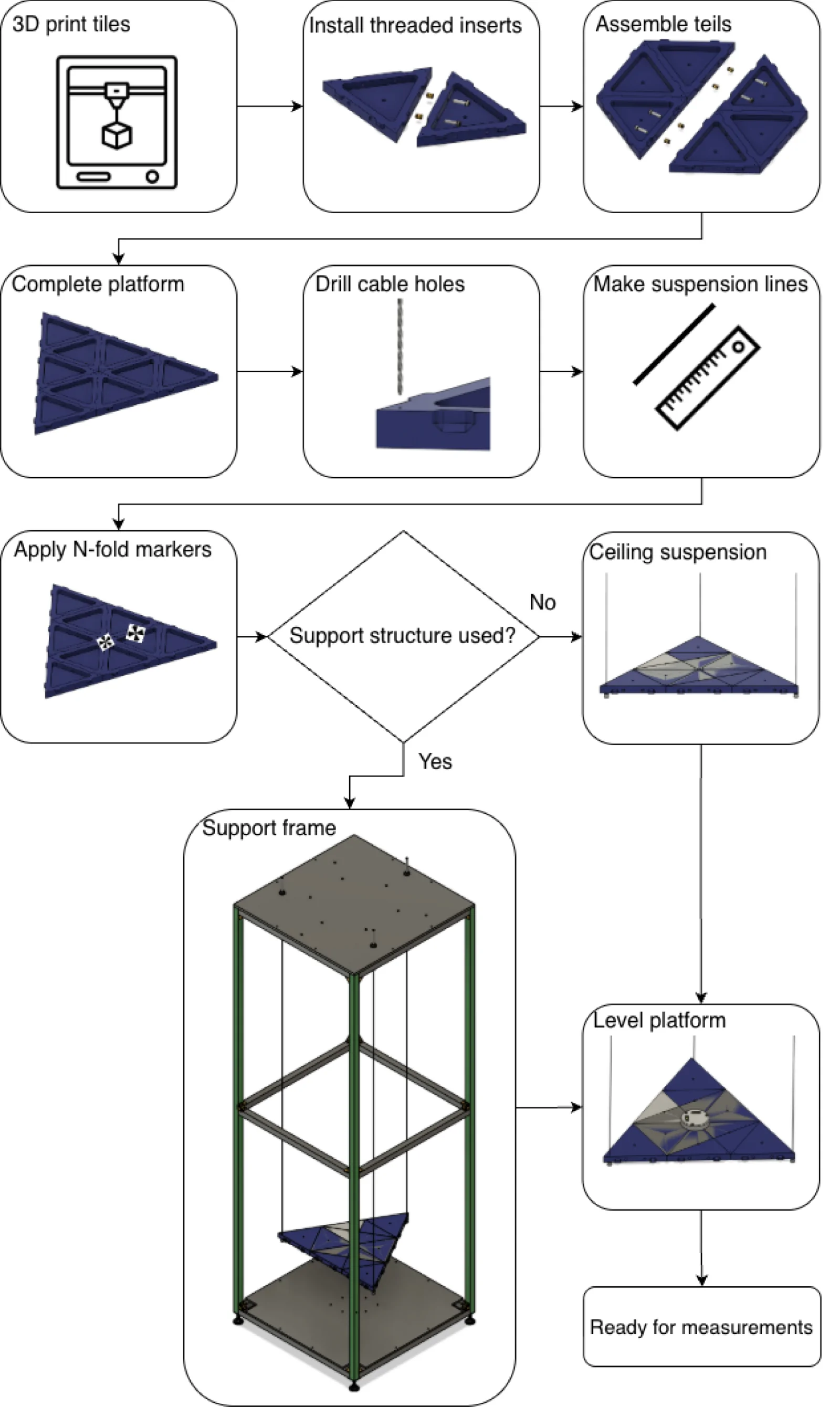
Assembly workflow of the proposed trifilar pendulum platform from fabrication of the printed tiles to the completed measurement system.

Fig. 4 provides an overview of the complete assembly process. The individual assembly steps are described in detail in the following subsections.

A complete set of build instructions, including the assembly guide for the support structure, is available in the online repository at https://doi.org/10.17632/zww548rfbn.6. The platform is the most critical component of the trifilar pendulum, as it can be used independently of the support structure if suitable attachment points are available. The full test setup, including the camera, platform, cables, purpose-built (optional) support structure, and the item under test (IUT), is shown in Fig. 2.

**Fig. 5:**
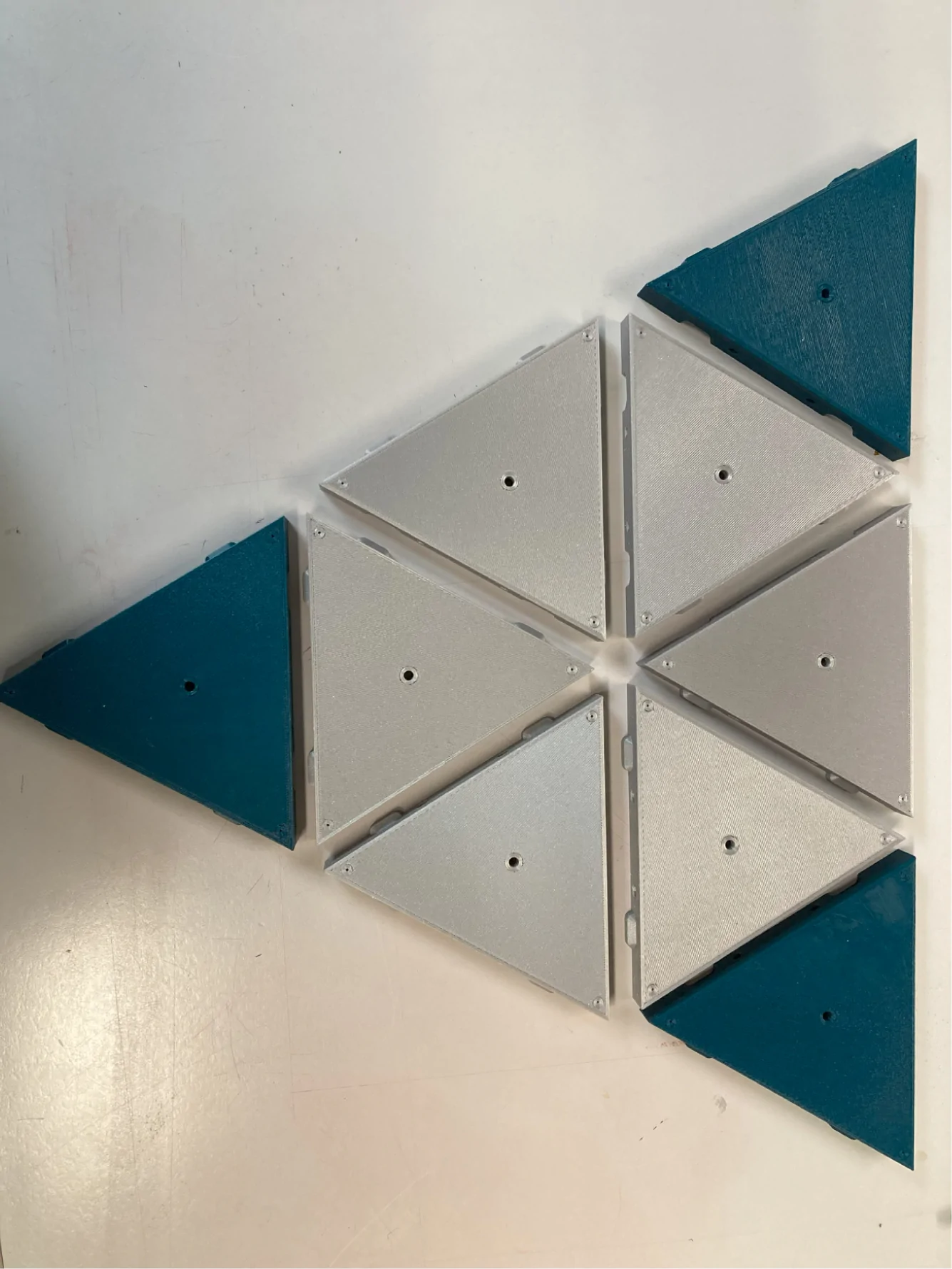
Platform pre-assembly check.

The recommended extrusion printer settings are provided in the detailed assembly instructions, and in Table 3 . It is recommended to first print a single triangular tile (Part #1) to evaluate and verify the print settings. If the result is satisfactory, two tiles can be printed simultaneously on a 200 mm × 200 mm print bed. Once all tiles are printed, the platform can be assembled by laying them out on a flat work surface to form a large triangle, as illustrated in Fig. 5.

**Fig. 6:**
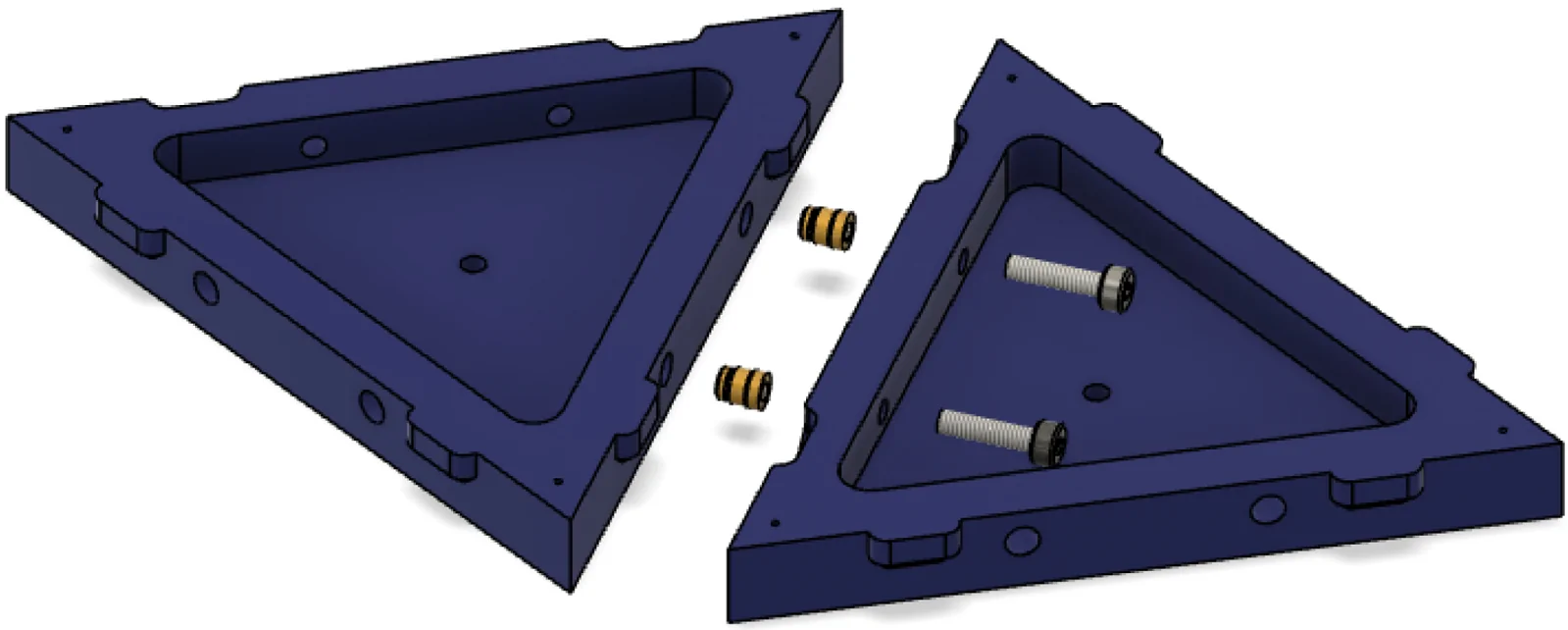
Assembly of two platform tiles.

Adjacent tiles are connected using two thermal threaded inserts (Part #2) in one tile and two M4 × 16 mm screws (Part #6) inserted through the adjacent tile and into the inserts, as illustrated in Fig. 6. To install the thermal brass inserts, place them into the designated holes and gently press them down until flush with the surface of the tile using a soldering iron set to 340 ° C. A thermal insert press can be used to achieve a more precise fit [18], of which there are open-source alternatives available. After insertion, press a flat surface against the insert and the surrounding PLA to prevent bulging of the material around the edges. Care should be taken not to touch the insert or surrounding PLA until it has cooled.

**Table 3:** Recommended slicing parameters used for fabrication of the platform tiles.

Parameter	Value
Slicer software	Cura
Printer	Prusa I3MK3
Material	PLA
Filament diameter	1.75 mm
Layer height	0.20 mm
Nozzle temperature	210 ° C
Build plate temperature	60 ° C
Wall count	4 perimeters
Infill density	25%
Infill pattern	honeycomb
Brim	Yes
Supports	None

The tiles are secured by fastening the M4 × 16 mm screws through one tile into the inserts of the adjacent tile, as shown in Fig. 6. This process is repeated for all tiles following the assembly strategy described below.

**Fig. 7:**
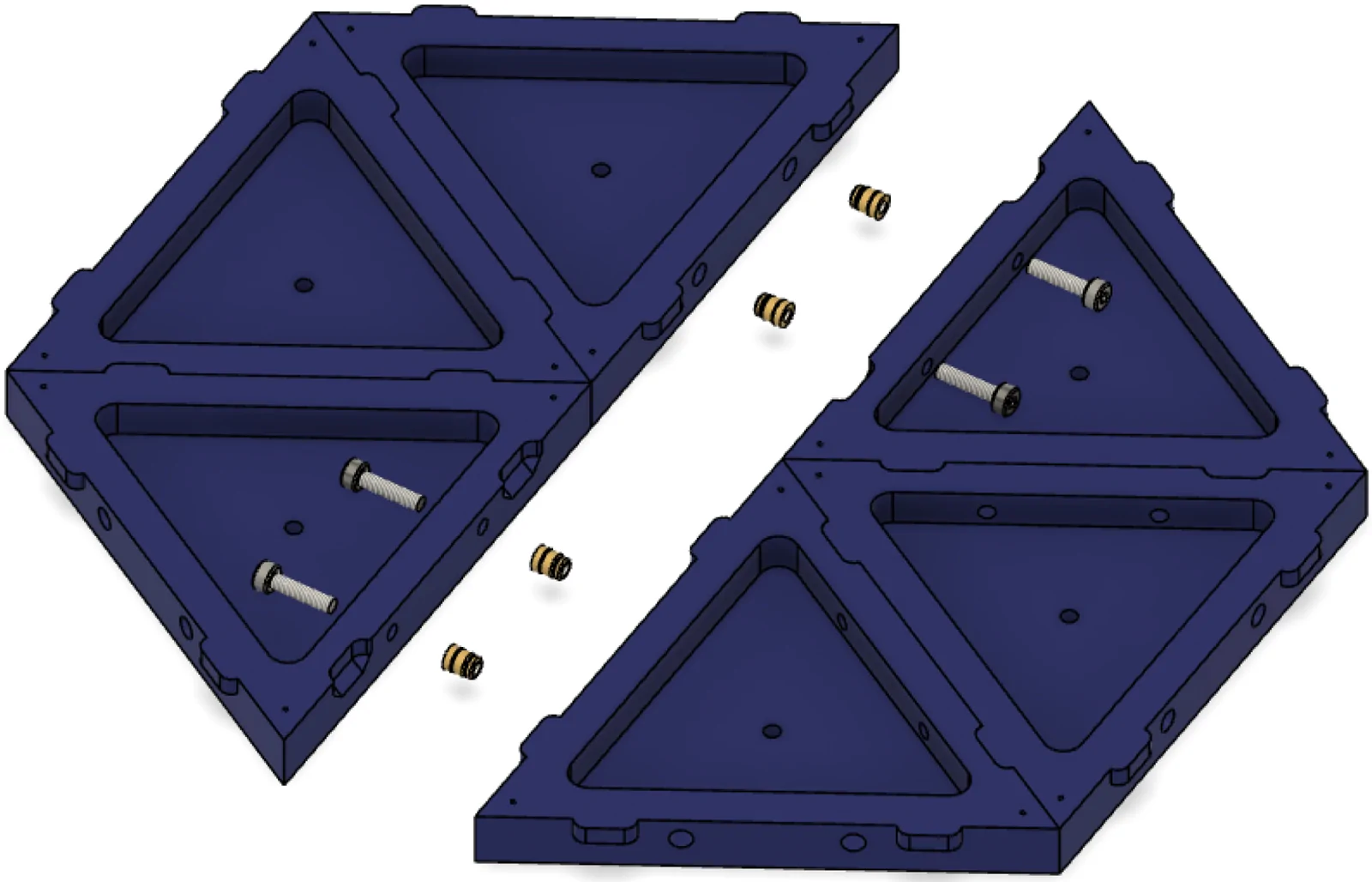
Sub assembly of platform.

**Fig. 8:**
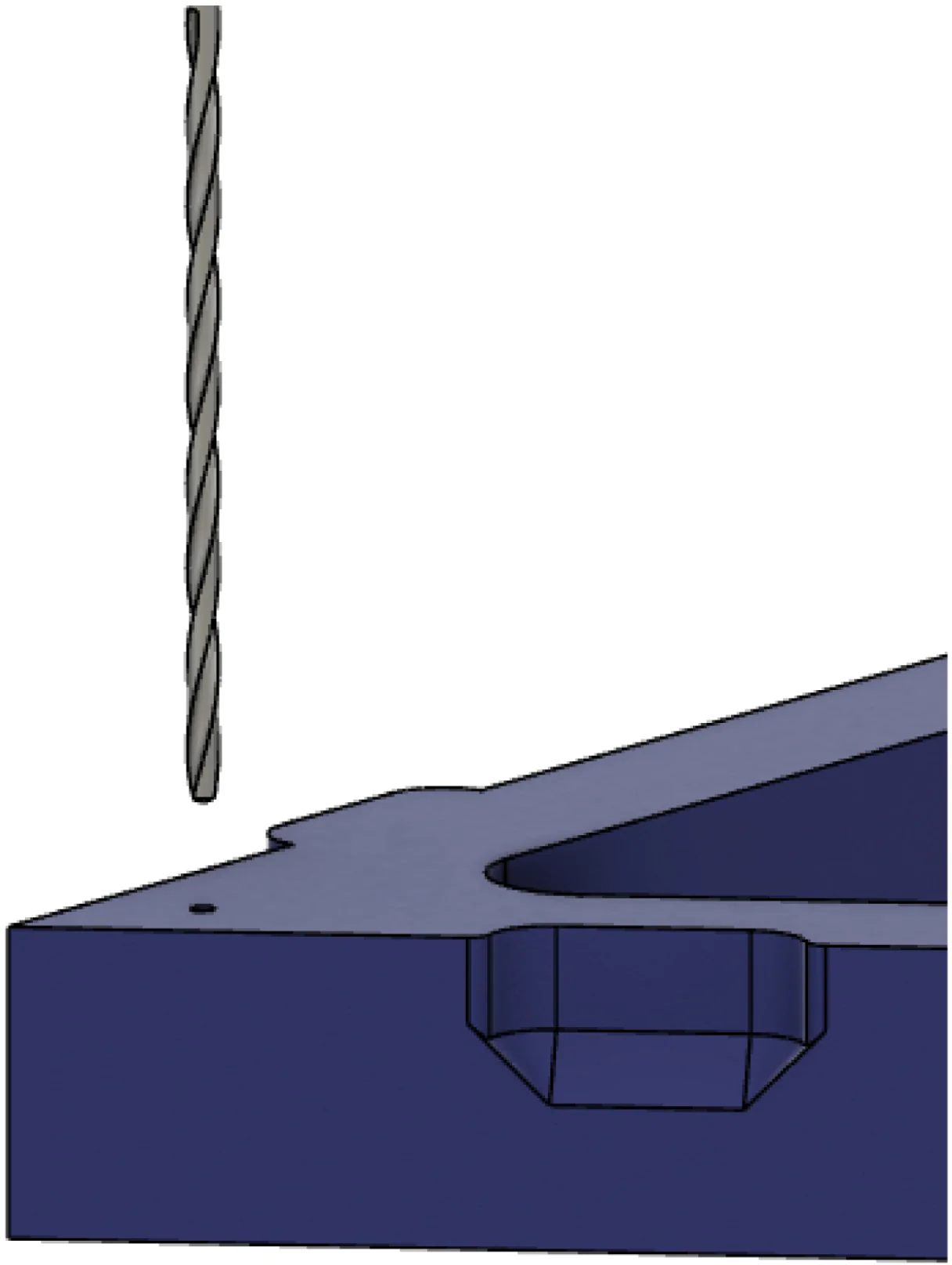
Drill the holes for cable attachment.

**Fig. 9:**
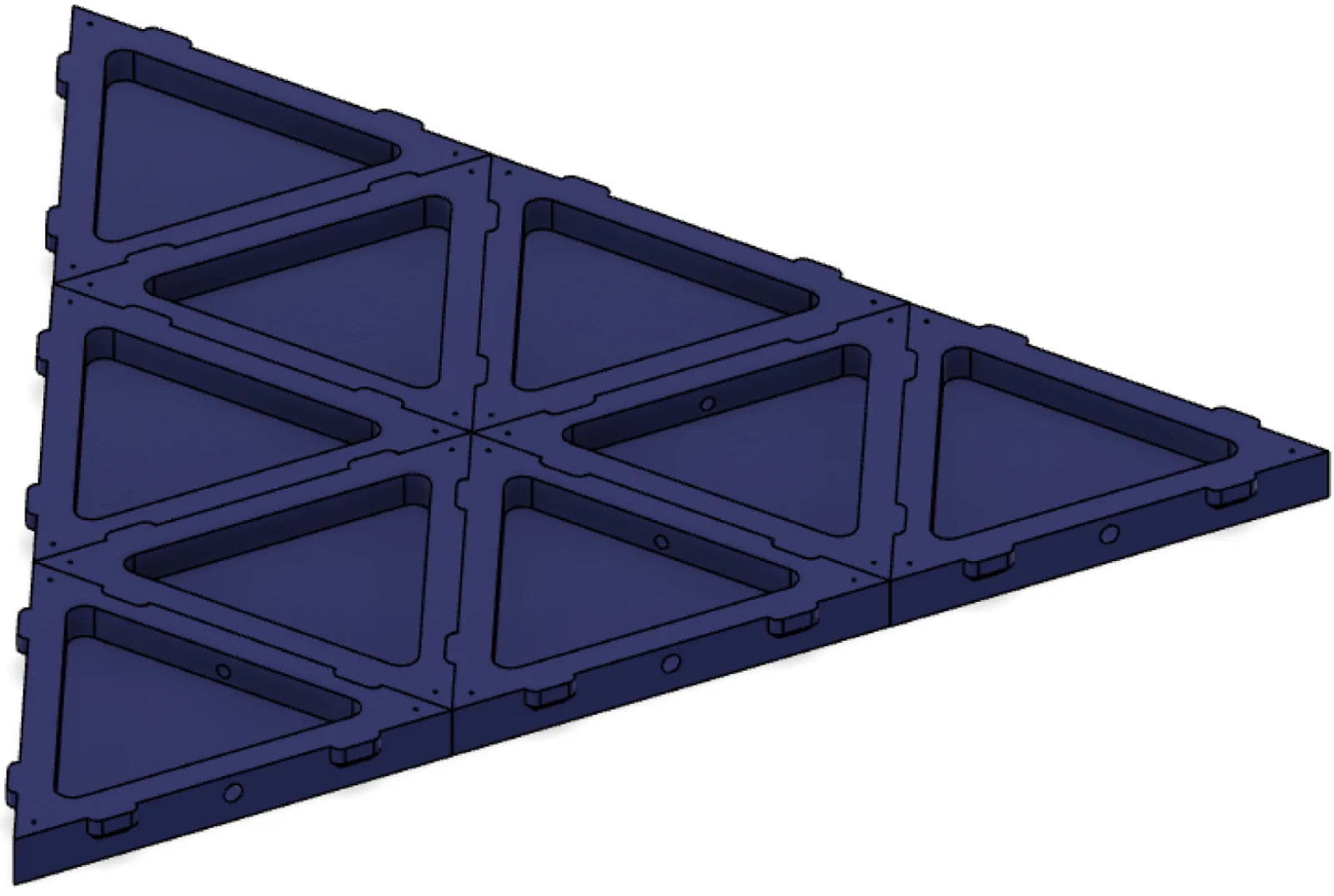
Assembled platform comprising 9 elements.

It is recommended to first assemble two sets of three tiles and then connect them to form the hexagonal shape shown in Fig. 7. Once the hexagon is constructed, determine the placement of the remaining three tiles before inserting the M4 thermal inserts (Part #2) to complete the final triangular form. Attach the last three tiles as shown in Fig. 9. For the final step, use a 1.5 mm drill bit to open the reinforced hole markings at each corner of the completed triangular platform, as illustrated in Fig. 8.

When making the suspension cables, be careful to ensure that they are of equal length. The recommended process is to mark a distance of 1350 mm on a flat work surface using masking tape. Measure 50 mm in from both ends of the 1350 mm line, so that the two inner tape markers are 1250 mm apart. Lay the cable (part # 3) between these marks and cut it to 1350 mm, securing one end with tape if needed. Before cutting the next cable, transfer the inner tape mark to the cable using a permanent marker. Repeat this for all three cables. The effective suspension length, L=1250mm, is defined as the distance between the inner reference marks on the suspension cables, corresponding approximately to the distance between the effective upper and lower pivot points. The additional cable length beyond these marks is required for attachment of the stop fittings and is not included in the value of L used in the calculations.

Next, attach the stop fittings (part # 4) to the cables at the marked points. Thread each cable through the small hole located at the points of the triangular platform. The platform can now be suspended from an existing structure (ceiling) or the frame, with detailed assembly instructions available on https://doi.org/10.17632/zww548rfbn.6. Two suspension lines materials have been considered for this project, steel wires and Dyneema®lines where the dyneema®are susceptible to abrasion and damage when in contact with sharp edges. Care should therefore be taken when testing valuable hardware. The connection points on the platform should be inspected and if necessary de-burred. The suspension radius R is measured as the distance between the geometric centre of the platform and the centre of each cable attachment hole. The attachment points are defined directly in the CAD model and may be verified using callipers during assembly. Prior to performing measurements, it is recommended to verify that the platform hangs level, that all three suspension lines are under tension and approximately vertical, and that the platform does not exhibit noticeable tilt or twist when at rest. The lengths of the suspension lines should be checked to ensure that the platform remains parallel to the floor. Any significant asymmetry should be corrected before measurements are performed.

## 6 Operation instructions

Detailed instructions for installing and using the software are available in the project repository https://doi.org/10.17632/zww548rfbn.6 and on the GitHub page [16]. Users are encouraged to modify and improve the setup for the benefit of others.

The measurement of MoI relies on the relationship shown in Eq. (1) [5] which can be derived through dynamics analysis of the system. The derivation of Eq. (1) assumes small-angle oscillations (sinθ≈θ), a rigid platform, equal-length massless and inextensible suspension lines, symmetric suspension geometry, negligible friction and negligible aerodynamic damping. It relates the MoI to the period of oscillation of the trifilar pendulum and fixed system parameters. I is the MoI around the axis of oscillation, g is the local acceleration due to gravity, m is the combined mass of the platform and Item Under Test (IUT), R is the distance from the platform’s centre to the wire attachment point, τ is the period of the oscillations and L is the cable length. Eq. (2) is used to calculate the MoI of the IUT, therefore the first step is to calculate the MoI of the platform, to obtain $I_{platform}$ (if not already known). Subsequent tests can then be performed with the IUT on the platform to obtain $I_{platform+IUT}$. When using the provided software Eqs. (2), (3), (4) will be applied to calculate the MoI automatically.

(1) $$I=\frac{g\cdot m\cdot R^{2}\cdot \tau^{2}}{4\cdot \pi^{2}\cdot L}$$

All quantities are expressed in SI units, with g in m s^−2^, m in kg, R and L in m, τ in s, and the resulting inertia I in kg m^2^. Internally, the software performs all calculations using SI units and reports the result in kg m^2^.

(2) $$I_{IUT}=I_{platform+IUT}-I_{platform}$$

(3) $$I_{IUT}=\frac{g\cdot m_{platform+IUT}\cdot R^{2}\cdot \tau_{platform+IUT}^{2}}{4\cdot \pi^{2}\cdot L}-\frac{g\cdot m_{platform}\cdot R^{2}\cdot \tau_{platform}^{2}}{4\cdot \pi^{2}\cdot L}$$

(4) $$I_{IUT}=\frac{g\cdot R^{2}}{4\cdot \pi^{2}\cdot L}\left(m_{platform+IUT}\cdot \tau_{platform+IUT}^{2}-m_{platform}\cdot \tau_{platform}^{2}\right)$$

The N-fold markers are attached to the underside of the platform using double-sided adhesive tape and pressed firmly against the surface to prevent movement during testing. Default settings put the N=5 marker at the centre of the platform and the N=4 marker approximately 10 cm away from the centre, depending on the camera used, it is important to make sure both markers stay in the field of view of the camera for the entire duration of the test.

**Fig. 10:**
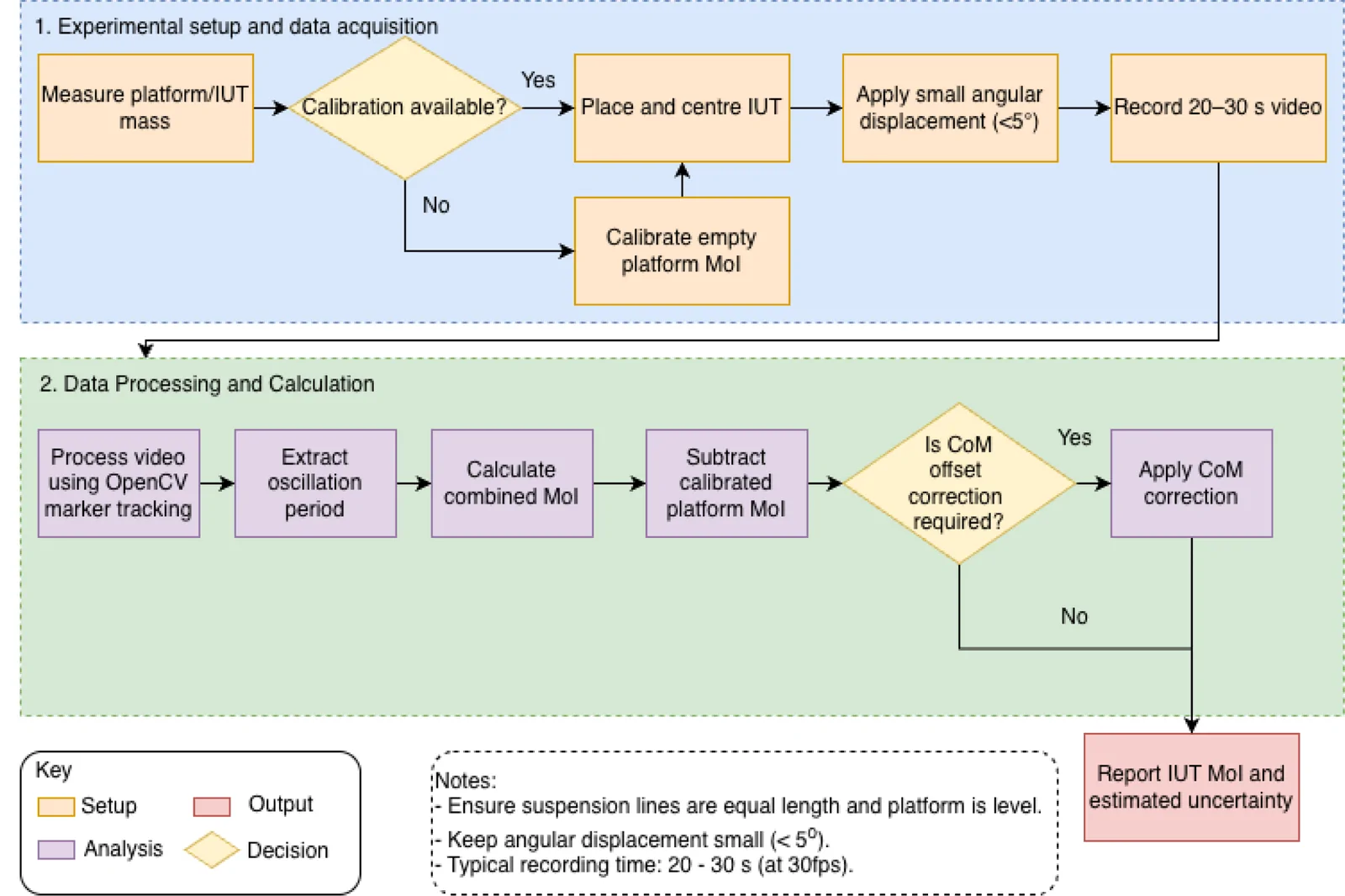
Complete measurement and data-processing workflow for determination of the mass moment of inertia of an item under test (IUT), from initial setup and platform calibration to video acquisition, optical marker tracking, period estimation, and calculation of the final IUT MoI.

In practice, the project software first determines the MoI of the empty platform using a dedicated calibration mode. The resulting value is stored and automatically loaded in subsequent measurements, allowing the software to compute $I_{platform+IUT}$ from the measured oscillation period and subtract the stored platform value to obtain $I_{IUT}$. The calibration value is stored in a file (calibration.json) and automatically loaded in subsequent measurements. The complete measurement and data-processing workflow is summarised in Fig. 10, followed by the detailed data processing and calculation

A step-by-step approach on how to conduct a measurement is now described:

Step 1 **Measure the mass of the test object**: Ensure that the mass of the object is known. The mass of the platform must also be known so it can be entered into the software during calibration.
Step 2 **Ensure platform is level, and check Camera position**: check if the platform is level to ensure equal cable tension. Ensure both N-fold markers are in the field of view of the camera (leaving some margin for pendulum motion during measurement).
Step 3 **Place the object on the platform**: Ensure its CoM is aligned with the platform’s centre (In case of a calibration run skip this step).
Step 4 **Apply a small angular displacement**: Gently rotate the platform by approximately 5° about the vertical axis (axis of oscillation) and release it smoothly, avoiding lateral sway. The excitation angle should remain below 10° to satisfy the small-angle assumption used in the MoI calculation.
Step 5 **Start the camera recording**: The camera should be positioned below the platform with the optical axis perpendicular to the platform plane and should operate at a fixed frame rate.
Step 6 **Save the video:** Preferably in .MP4 format for further processing.
Step 7 **Repeat measurement:** It is recommended to perform five repeated measurements for each test condition to reduce the influence of random measurement variability.
Step 8 **Change orientation of IUT**: To measure a different MoI of the same object its orientation can be changed, the above steps should be repeated for this new orientation. (In case of a calibration run skip this step)

In practice, perpendicular camera alignment is achieved by positioning the camera approximately beneath the platform and visually centring the two N-fold markers within the image. Small deviations from perfect perpendicularity were found to have negligible influence on the measured oscillation period. By using two markers the software is able to account for any sway introduced by the operator, however an effort should be made to reduce the sway of the platform as much as possible in order to avoid the markers from leaving the image frame

### 6.1 Software and data handling

The image-processing software is provided in the project repository and can be executed locally after downloading the source code and installing the required Python packages listed in the accompanying requirements.txt file, of which a detailed summary is shown in Table 4. The software was tested on macOS using Python 3.9.6 and on Windows using Python 3.11.0. On macOS, Python and package installation commands may need to be called using python3 and pip3, whereas on Windows the commands python and pip are typically used.

After navigating to the software directory in a terminal, the required packages can be installed using:

pip3 install -r requirements.txt on macOS, or:

pip install -r requirements.txt on Windows.

To verify that the Python environment, OpenCV installation, and analysis pipeline have been installed correctly, a command-line self-test can be executed before using the graphical interface. The self-test processes the supplied reference video (self_test_video.mp4) using the same image-processing pipeline as the GUI and compares the calculated oscillation period and MoI against a validated reference dataset. Successful completion confirms that the software installation is functioning as intended and provides users with a reproducible baseline before analysing their own measurements. The self-test can be started using:

python3 self_test.py on macOS, or:

python self_test.py on Windows.

The MoI calculation software can then be launched from the terminal using:

python3 Moi_calculation_automatic.py on macOS, or:

python Moi_calculation_automatic.py on Windows.

Upon execution, the user is presented with the graphical user interface shown in Fig. 11. The user is prompted to select the input video file, specify an output folder, and enter the mass of the platform and the mass of the item under test. If the user has deviated from the design recommendations presented in this paper, alternative suspension length and platform radius values can be entered in the corresponding fields. The marker size and video frame rate can also be adjusted if required; however, when the recommended design parameters are used, the default values do not need to be changed. Processing is initiated by pressing the *Process* button. The MP4 video file then opens in a separate window, where the detected tracking markers are displayed during playback. Once the video has been processed, this window closes automatically and the calculated MoI, oscillation period, estimated excitation angle, estimated measurement error, and motion plot are displayed in the graphical user interface, as shown in Fig. 11. Depending on the size of the MP4 file, a short delay may occur before the results are displayed. Processing can be interrupted at any time by pressing the q key.

**Table 4:** Python packages that are installed using the requirements.txt file.

Library	Version
Python	3.9.6/3.11.0
OpenCV	>=4.7
NumPy	>=1.2
Pandas	>=1.4
PyQt6	>=6.5
moviepy	>=2.0
matplotlib	>=3.6

When a measurement is finished, the user can select a new video file in the GUI, adjust the mass (if required) and start a new measurement. A new output file is automatically created for each measurement using a timestamp with sub-second resolution, preventing filename conflicts when measurements are processed in rapid succession.

An example test procedure as well as sample data can be found on the online repository https://doi.org/10.17632/zww548rfbn.6.

A recording of 20 to 30 s is advised for every test to have sufficient oscillations for measurement of the period. The goal is to capture a minimum of 10 oscillations. The software will average the oscillation period measured over the individual measured oscillations. If the video is too long, measurements may be negatively influenced due to damping in the system shifting the oscillation frequency of the pendulum.Fig. 11User interface for video processing. Image of the user interface once the processing is complete, showing the MoI, period and Oscillation plot. In image b the recording is shown, with the N-fold markers and the targets overlayed in purple.Fig. 11

**(a):**
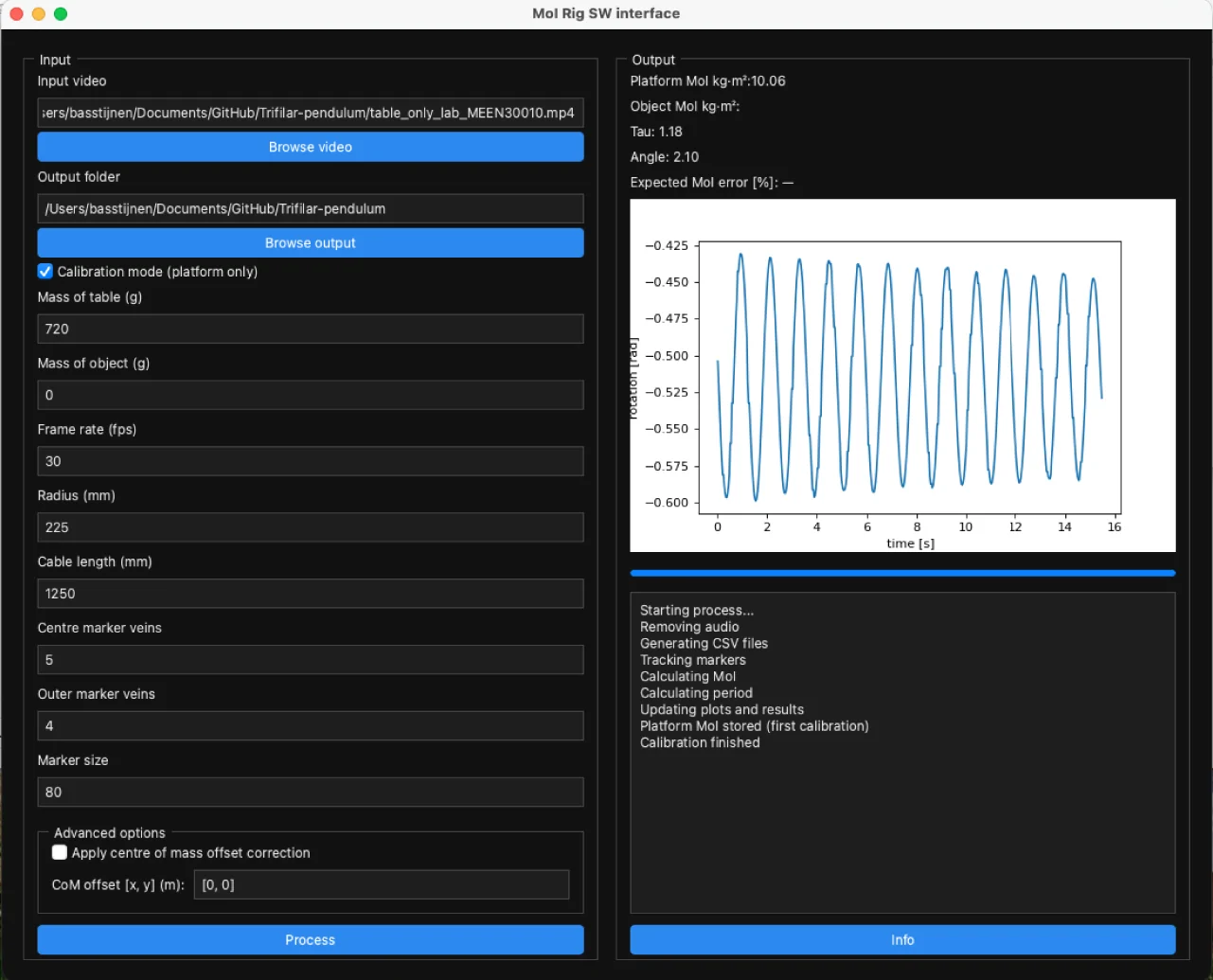
.

**(b):**
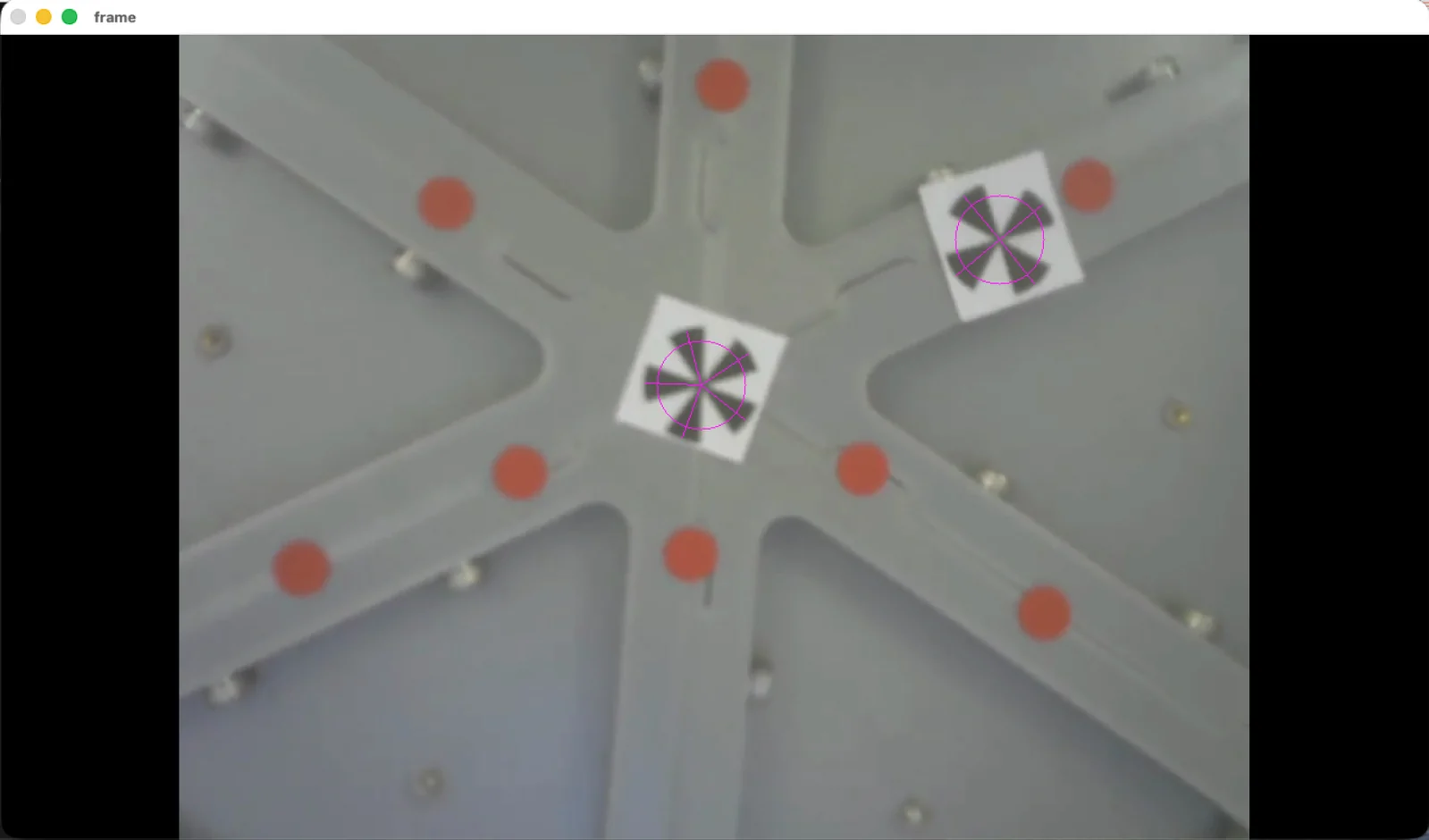
.

### 6.2 Centre of mass offset correction and error estimation

The theoretical formulation of the trifilar pendulum assumes that the centre of mass (CoM) of the item under test (IUT) is aligned with the geometric centre of the platform. In practice, achieving perfect alignment can be challenging, particularly for irregularly shaped objects or assemblies with non-uniform mass distributions. A lateral offset of the CoM introduces an additional contribution to the measured MoI. To account for this effect, the accompanying software includes an optional centre of mass offset correction. When enabled by the user, the offset of the IUT CoM relative to the platform centre is specified in the plane of the platform. The correction is applied using the parallel axis theorem, such that the MoI about the IUT CoM is given by Eq. (5)

(5) $$I_{\mathrm{CoM}}=I_{\mathrm{measured}}-m\cdot d^{2}$$

In Eq. (5) m is the mass of the IUT and d is the radial distance between the platform centre and the IUT CoM. The correction depends only on the magnitude of the offset, and is independent of its orientation in the plane of the platform. The centre of mass offset input is optional and disabled by default. If no offset is specified, the software reproduces the original measurement behaviour. This approach preserves ease of use for standard measurements while allowing advanced users to compensate for known alignment errors. In cases where the specified CoM offset is inconsistent with the measured inertia, the correction may result in a non-physical (negative) MoI. For example, specifying a large CoM offset for a low-inertia object may result in a correction term larger than the measured inertia, producing a negative value. Such results should be interpreted as evidence of inconsistent input parameters or experimental assumptions. The software detects this condition and provides a warning to the user, indicating that one or more input parameters or experimental assumptions may be invalid. Such cases serve as a diagnostic indicator of measurement inconsistency rather than a physically meaningful result. The implemented correction considers only in-plane CoM offsets. Vertical offsets relative to the platform plane are not compensated. However, for typical CubeSat and small spacecraft test configurations the vertical offset is generally small compared with the suspension length and its influence is therefore expected to be limited. Users testing tall or strongly asymmetric objects should take this limitation into account.

In addition to the CoM correction, the software provides an estimate of the expected relative measurement error based on first-order uncertainty propagation applied to the trifilar pendulum relation. The estimate accounts for uncertainties in the measured mass, suspension radius, suspension length, and oscillation period. For each measurement, the corresponding sensitivity coefficients are obtained from the analytical partial derivatives of the inertia equation with respect to these quantities, and the combined uncertainty is calculated using a root-sum-square approach. The resulting inertia uncertainty is then normalised by the calculated MoI of the item under test to obtain an estimated relative error.

(6) $$\sigma_{I}=\sqrt{{\left(\frac{\partial I}{\partial m}\sigma_{m}\right)}^{2}+{\left(\frac{\partial I}{\partial R}\sigma_{R}\right)}^{2}+{\left(\frac{\partial I}{\partial L}\sigma_{L}\right)}^{2}+{\left(\frac{\partial I}{\partial \tau }\sigma_{\tau }\right)}^{2}}$$

**Table 5:** Uncertainty values used by the software to estimate error.

Parameter	Value	Source
$\sigma_{m}$	0.001 kg	kitchen scale resolution
$\sigma_{R}$	1 mm	ruler/tape measure
$\sigma_{L}$	1 mm	ruler/tape measure
$\sigma_{\tau }$	0.01 s	period estimation

In Eq. (6), $\sigma_{m}$, $\sigma_{R}$, $\sigma_{L}$, and $\sigma_{\tau }$ are the assumed uncertainties in mass, suspension radius, suspension length, and oscillation period, respectively and which are shown in Table 5. Eq. (6) assumes that the uncertainty sources are uncorrelated. Although small correlations may exist between geometric measurements such as R and L when obtained using the same measuring instrument, these effects are expected to be negligible compared with the overall uncertainty budget and are therefore ignored. The expected relative measurement error is then estimated using Eq. (7).

(7) $$\epsilon_{\mathrm{rel}}=\frac{\sigma_{I}}{I_{\mathrm{IUT}}}$$

In Eq. (7), $I_{\mathrm{IUT}}$ is the calculated mass moment of inertia of the item under test. This value is reported alongside the measured MoI as a practical indication of measurement sensitivity and reliability, rather than as a formal metrological uncertainty budget.

## 7 Validation and characterisation

To assess the accuracy and robustness of the proposed experimental setup, a validation study was conducted with particular emphasis on the data acquisition method and suspension configuration. Previous studies by Du Bois [5] and Titchener [13] have performed extensive error analysis on trifilar pendulum measurements using alternative instrumentation.

The validation experiments examine the influence of:

• camera type and frame rate,
• camera distance from the platform,
• suspension cable material,
• and the use of a purpose-built support structure compared to ceiling suspension.

Together, these tests provide insight into the sensitivity of the measurement method to practical experimental parameters and demonstrate the robustness of the proposed low-cost measurement system.

The MoI of the empty platform was measured experimentally as approximately $7.996\times 10^{-3}\;\mathrm{kg}\;m^{2}$ with a standard deviation of $1.3240\times 10^{-5}\;\mathrm{kg}\;m^{2}$ (0.17%) obtained from five repeated measurements. For comparison, the CAD model predicts a platform MoI of $7.296\times 10^{-3}\;\mathrm{kg}\;m^{2}$, corresponding to a difference of approximately 9.6%. The CAD model includes the 3D printed tiles, brass threaded inserts, and stainless-steel fasteners used in the assembly. However, the model assumes a homogeneous material distribution within each 3D-printed component based on the specified material density (experimentally determined by weighing one of the 3D printed tiles). In practice, fused filament fabrication produces a non-uniform internal structure consisting of perimeter walls, solid top and bottom layers, and infill patterns, which may alter the actual mass distribution and resulting MoI. In addition, the experimentally measured value includes contributions from the suspension lines and attachment hardware used during calibration, which were not represented in the CAD model. For these reasons, the experimentally determined platform MoI was used in all subsequent calculations.

Comparative tests were conducted using objects with analytically known MoIs. The validation objects consisted of circular calibration masses with a central through-hole, allowing them to be mounted concentrically or at prescribed radial offsets on the platform. Two types of calibration masses were used, denoted 410 g and 811 g. Different theoretical MoI cases were generated by varying the number of masses and their radial placement relative to the platform centre. In configurations with multiple masses, the weights were arranged symmetrically about the platform centre so that the combined centre of mass remained aligned with the axis of rotation while the overall MoI was varied. For example, some test cases were produced by placing two identical masses at equal radial distances on opposite sides of the platform centre. The theoretical MoI of each configuration was calculated from the known mass and geometry of the calibration weights together with their radial positions, using the parallel axis theorem where applicable. The error between measured and theoretical values was computed using Eq. (8), where the measurement error is defined as the absolute difference between the measured MoI ($I_{test}$) and the theoretical MoI ($I_{theory}$), normalised by the theoretical value of the IUT.

All tests were conducted according to a standardised measurement procedure available in the project database 10.17632/zww548rfbn.4. Prior to each experimental campaign, the MoI of the empty platform was determined using five calibration runs. For each subsequent test configuration, five measurements were recorded and the reported MoI value corresponds to the average of these measurements. The error bars shown in the figures therefore represent the spread between the minimum and maximum measured values. Each test configuration was measured five times. Repeated measurements were used to estimate the repeatability of the method and to reduce the influence of random measurement variability. While increasing the number of repetitions decreases the uncertainty of the mean approximately in proportion to $1/\sqrt{n}$, five repetitions were selected as a practical compromise between statistical confidence and experimental effort [19].

(8) $$Error=abs\left(\frac{I_{test}-I_{theory}}{I_{theory}}\right)\cdot 100$$

Across the validation experiments, nine different MoI values were evaluated, ranging from $0.15\cdot 10^{-3}\;\mathrm{kg}\;m^{2}$ to $6.1\cdot 10^{-3}\;\mathrm{kg}\;m^{2}$. For clarity, the observed performance is classified into three regimes: high error (E>15%), moderate error (5%≤E≤15%), and low error (E<5%) summarised in Table 6. These ranges provide a practical indication of the measurement accuracy that can be expected when using the proposed trifilar pendulum with camera-based tracking. These thresholds were selected empirically from the validation results and correspond approximately to increasing ratios between the inertia of the item under test and the inertia of the platform. As the IUT inertia becomes small relative to the platform inertia, subtraction of the platform contribution amplifies measurement uncertainty and increases sensitivity to modelling assumptions. However, the accuracy is also influenced by several experimental parameters, which are explored in the following subsections.

**Table 6:** Summary of performance metrics across the tested MoI range.

MoI range ($m^{2}\mathrm{kg}$)	Error performance
$I<10^{-3}$	E>15%
$10^{-3}\leq I\leq 3\cdot 10^{-3}$	5%≤E≤15%
$I>3\cdot 10^{-3}$	E<5%

### 7.1 Measurements

**Fig. 12:**
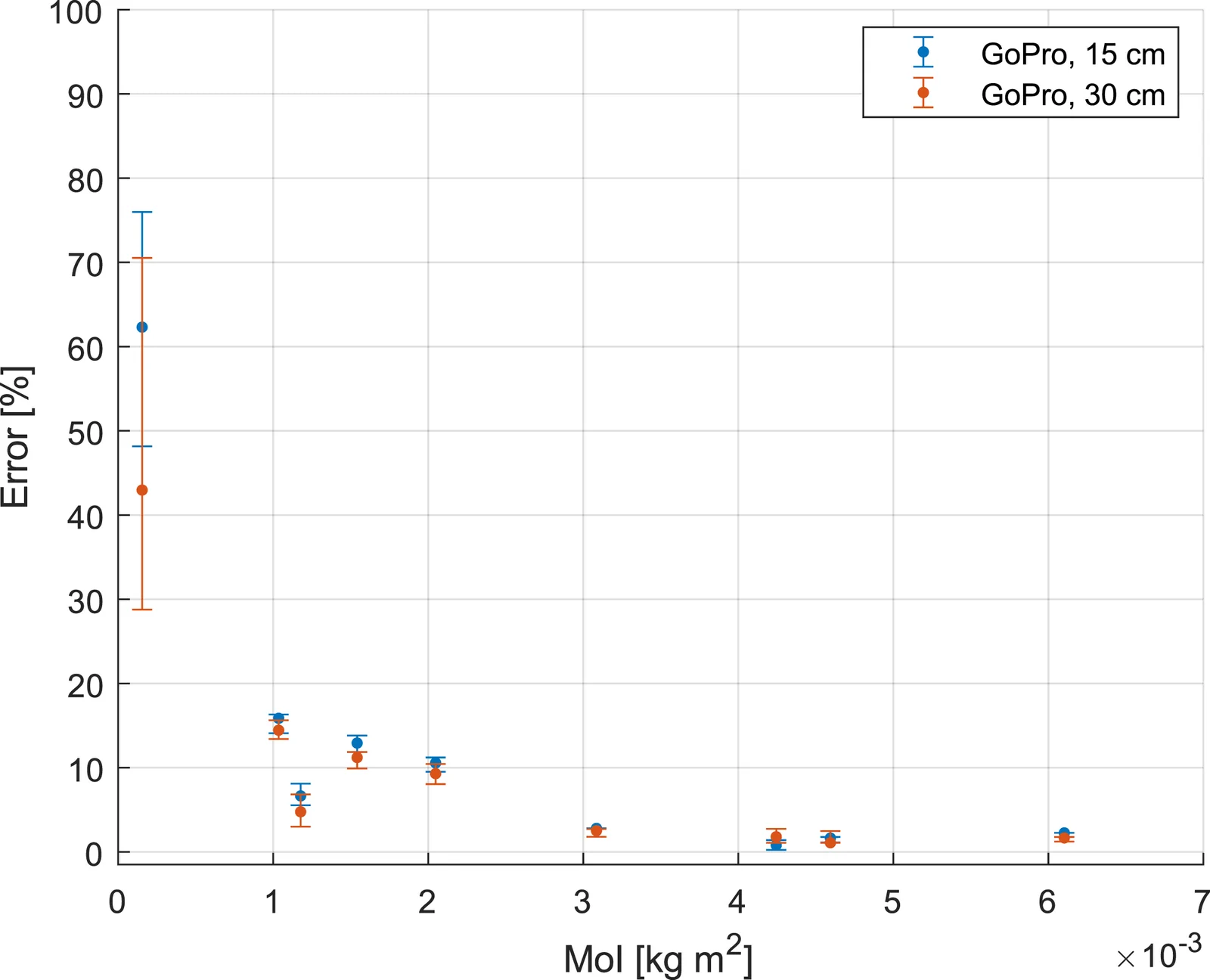
Tests with the platform suspended from the ceiling using steel cables, recorded with a GoPro 7 Black at 50 fps. The camera was positioned 30 cm and 15 cm below the platform.

**Fig. 13:**
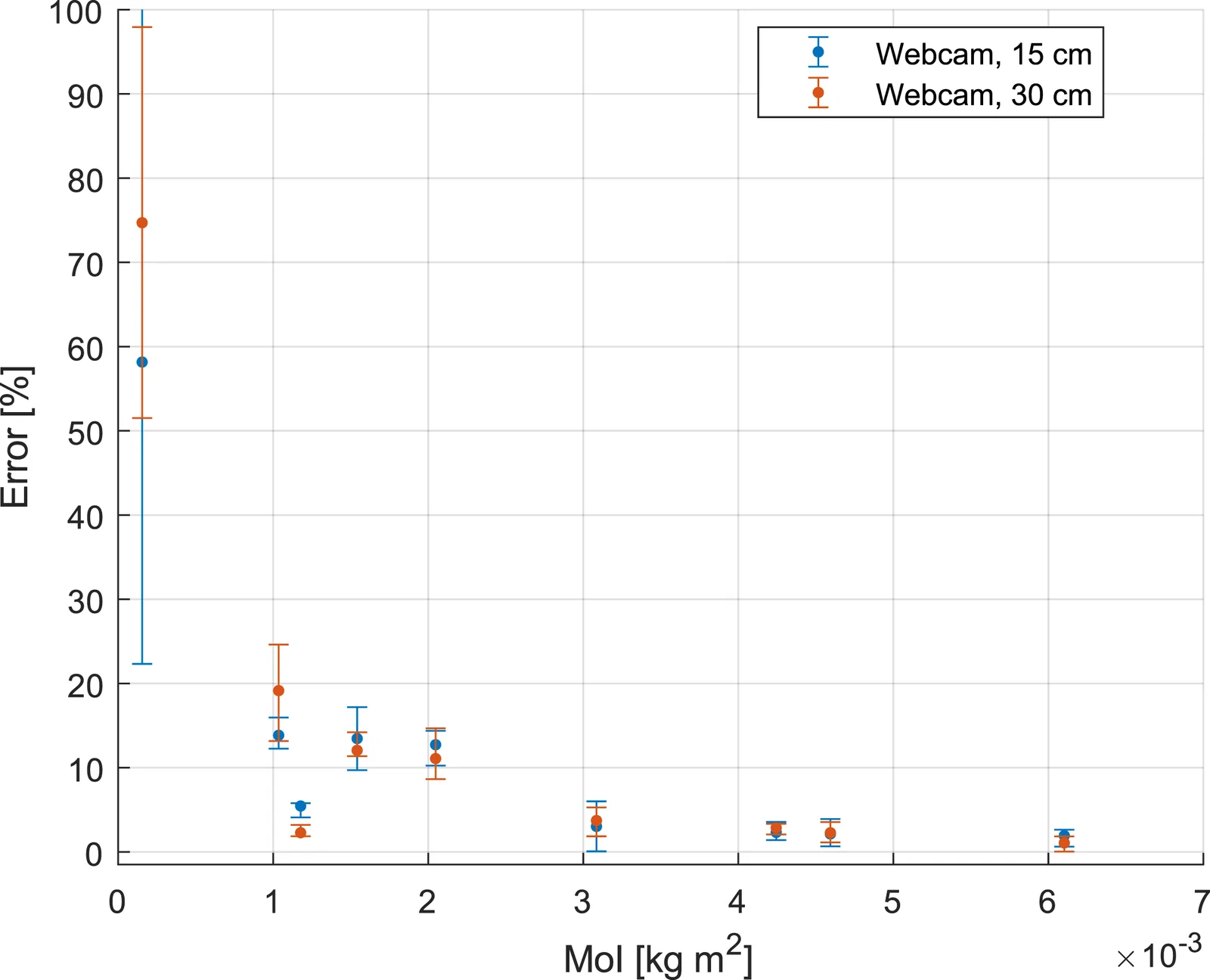
Tests with the platform suspended from the ceiling using steel cables. Data were captured using a Logitech™ C270 webcam at 30 fps with camera distances of 15 cm and 30 cm.

Fig. 12, Fig. 13 present the measurement errors obtained for nine MoI cases using two different cameras: a GoPro 7 Black [20] and a Logitech™ C270 webcam [21], a summary of the results can be found in Table 7, Table 8. In these tests, the platform was suspended from the ceiling using steel cables with metal stop fittings. The error bars represent the range between the maximum and minimum measured values across five trials, while the solid markers indicate the mean value. The GoPro 7 Black is capable of recording high-definition video (up to 4 K) at frame rates of up to 60 frames per second, with a wide-angle lens and high-quality image sensor, whereas the Logitech C270 webcam operates at a maximum resolution of 720p with a frame rate of 30 frames per second and more limited optical performance.

For MoI values greater than $10^{-3}\;\mathrm{kg}\;m^{2}$, both cameras exhibit similar performance. However, for MoI values below $10^{-3}\;\mathrm{kg}\;m^{2}$, the GoPro demonstrates approximately 10% lower measurement error compared to the webcam. This behaviour is attributed primarily to the higher frame rate and improved optical quality of the GoPro camera, which improves marker tracking resolution.

In addition to camera type, the effect of camera distance was also investigated. Measurements were conducted with the camera positioned 15 cm and 30 cm below the platform. The shorter distance represents the closest configuration that still allows both markers to remain within the limited field of view of the webcam. The results show minimal sensitivity to camera distance for MoI values between 10^−3^ and $3\cdot 10^{-3}\;\mathrm{kg}\;m^{2}$, with differences of only 1%–2%. For MoI values exceeding $3\cdot 10^{-3}\;\mathrm{kg}\;m^{2}$, the discrepancy becomes negligible, typically between 0.1% and 0.4%.

For MoI values below 10^6^, the GoPro results show a slightly higher error at the shorter camera distance. This counter-intuitive behaviour is attributed to fish-eye distortion introduced by the wide-angle lens, which becomes more pronounced when the markers approach the edges of the image frame.

**Fig. 14:**
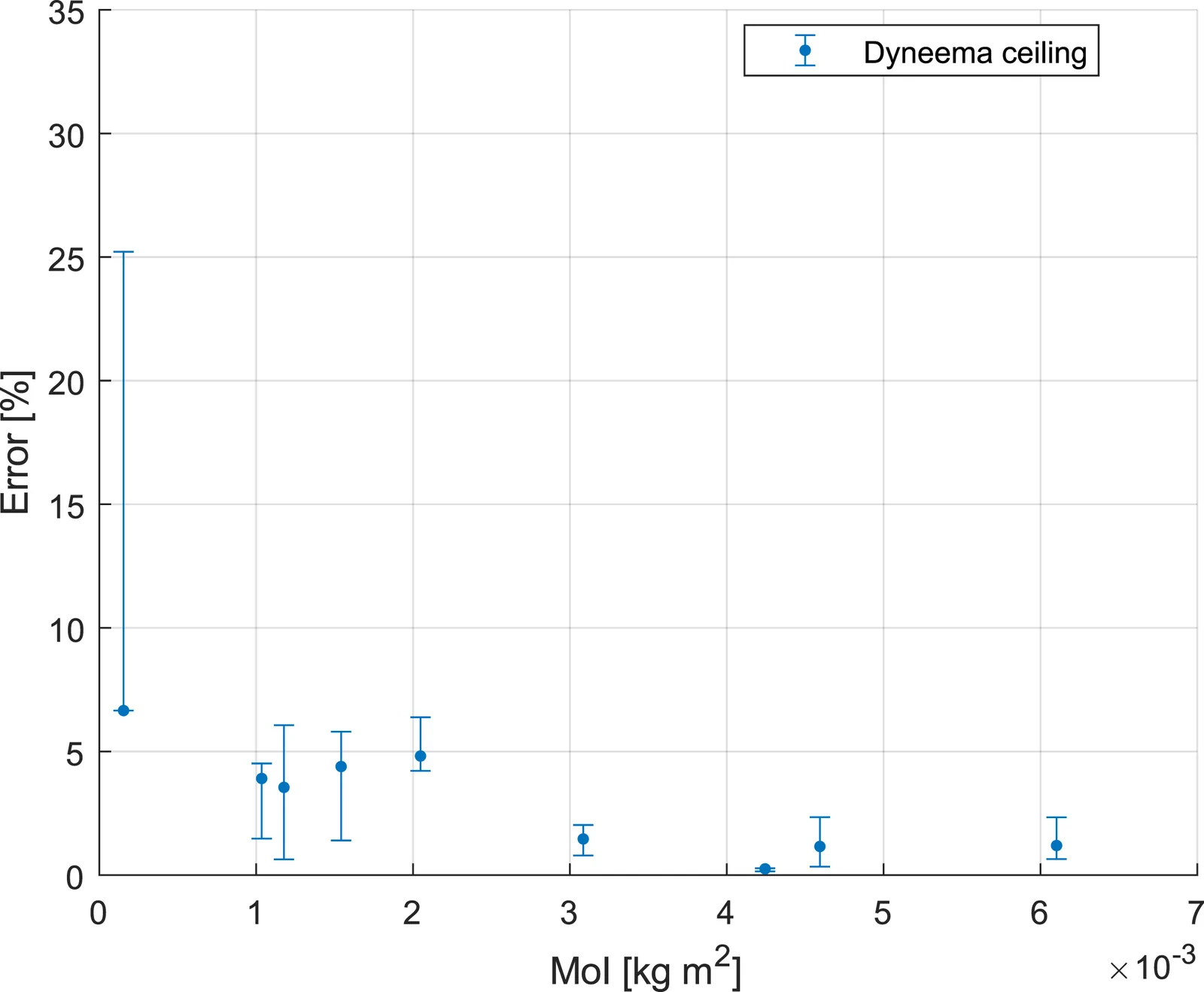
Tests with the platform suspended from the ceiling using Dyneema® lines.

In an effort to improve measurement accuracy for lower MoI values, the steel cables and cable stop fittings were replaced with Tergo® braided Dyneema® lines. The results of these tests are shown in Fig. 14 and summarised in Table 9. In this configuration, the platform remained suspended from the ceiling while measurements were recorded using the Logitech webcam positioned 15 cm below the platform.

**Table 7:** Tests with the platform suspended from the ceiling using steel cables, recorded with a GoPro 7 Black at 50 fps. The camera was positioned 30 cm and 15 cm below the platform. Every test was repeated 5 times.

Theoretical MoI ${\mathrm{kg m}}^{2}$	Mean measured MoI ${\mathrm{kg m}}^{2}$	Std measured MoI ${\mathrm{kg m}}^{2}$	Percent error
0.0001547	0.0002446	7.18e−05	58.16
0.0010353	0.0011786	2e−05	13.85
0.0011772	0.0012413	8.9e−06	5.45
0.0015415	0.001749	4.31e−05	13.46
0.0020478	0.0023084	4.64e−05	12.73
0.0030853	0.0031786	7.09e−05	3.03
0.0042447	0.0041462	3.87e−05	2.32
0.004594	0.004692	6.04e−05	2.13
0.0061028	0.0062208	5.17e−05	1.93

**Table 8:** Tests with the platform suspended from the ceiling using steel cables. Data were captured using a Logitech™ C270 webcam at 30 fps with camera distances of 15 cm and 30 cm. Every test was repeated 5 times.

Theoretical MoI ${\mathrm{kg m}}^{2}$	Mean measured MoI ${\mathrm{kg m}}^{2}$	Std measured MoI ${\mathrm{kg m}}^{2}$	Percent error
0.0001547	0.000251	1.53e−05	62.31
0.0010353	0.0011995	1.03e−05	15.86
0.0011772	0.0012554	1.27e−05	6.65
0.0015415	0.0017407	1.29e−05	12.92
0.0020478	0.0022642	1.29e−05	10.57
0.0030853	0.0031715	6e−07	2.79
0.0042447	0.0042108	2.06e−05	0.8
0.004594	0.0046694	1.33e−05	1.64
0.0061028	0.0062406	0	2.26

The use of Dyneema lines produced the best overall results for this pendulum configuration. For MoI values above $10^{-3}\;\mathrm{kg}\;m^{2}$, the measurement error remained below 5%, while for MoI values below $10^{-3}\;\mathrm{kg}\;m^{2}$ the error was reduced to approximately 6.6%. The results indicate that the use of Dyneema® suspension lines reduces the measurement error compared with the steel cable configuration, particularly for low-inertia test cases. Although the individual contributions were not quantified experimentally, the observed improvement is believed to result from differences in suspension dynamics, including cable mass, bending stiffness, torsional stiffness, and attachment behaviour. These factors influence how closely the physical system approximates the ideal trifilar pendulum model. Consequently, low-mass, flexible suspension lines such as Dyneema are recommended for future implementations of the system.

The previous experiment was repeated using the purpose-built support structure described in Section 4 instead of ceiling suspension. The goal of this experiment was to determine whether the structural frame introduces additional measurement error due to structural flexibility or vibration.

**Table 9:** Tests with the platform suspended from the ceiling using Dyneema® lines. Every test was repeated 5 times.

Theoretical MoI ${\mathrm{kg m}}^{2}$	Mean measured MoI ${\mathrm{kg m}}^{2}$	Std measured MoI ${\mathrm{kg m}}^{2}$	Percent error
0.0001547	0.0001444	2.03e−05	6.66
0.0010353	0.0010757	1.41e−05	3.91
0.0011772	0.0011354	2.93e−05	3.55
0.0015415	0.0016092	2.93e−05	4.39
0.0020478	0.0021464	1.84e−05	4.82
0.0030853	0.0031304	1.35e−05	1.46
0.0042447	0.0042554	2.4e−06	0.25
0.004594	0.0046473	4.97e−05	1.16
0.0061028	0.0061759	4.41e−05	1.2

**Fig. 15:**
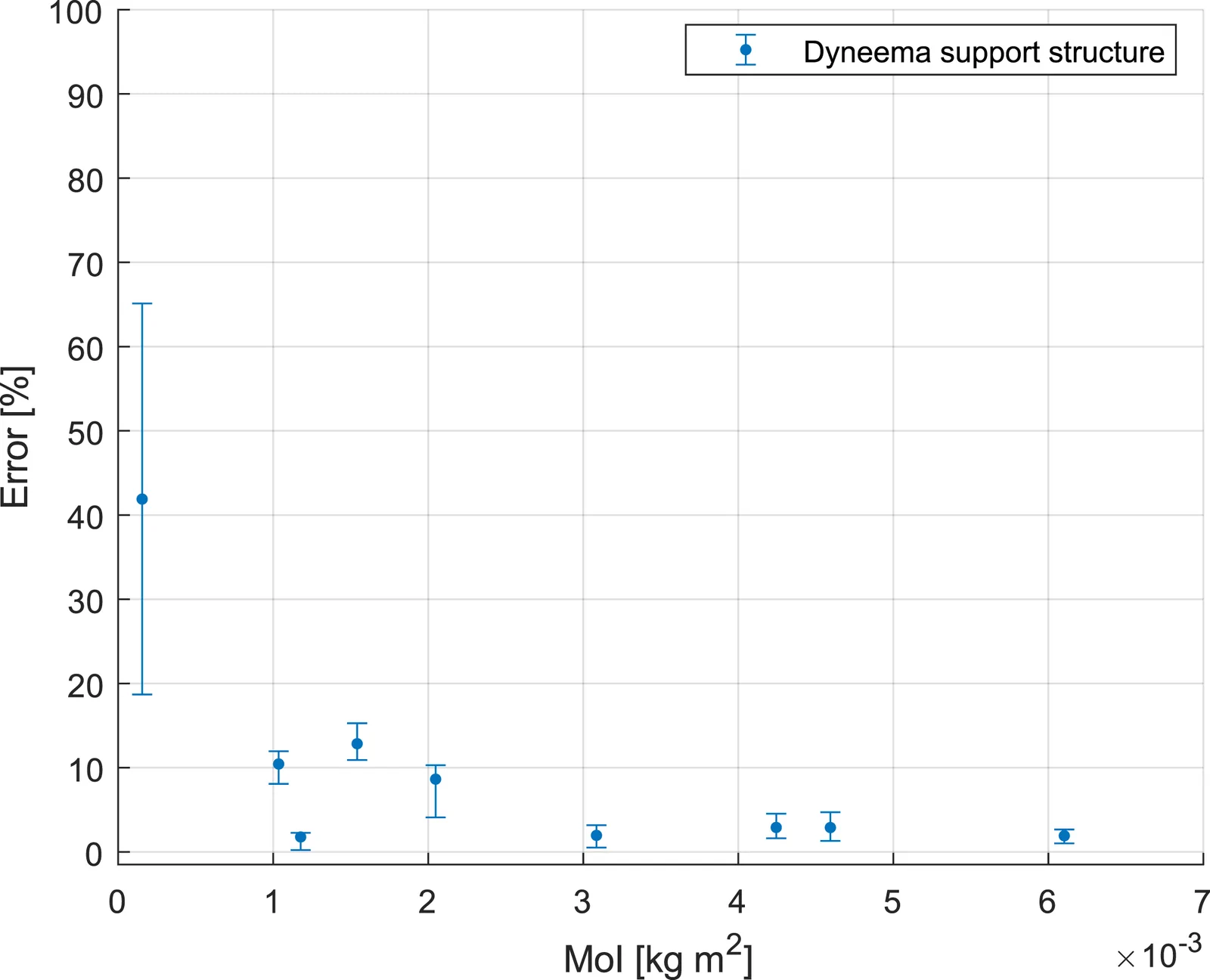
Tests with the platform suspended from the purpose-built support structure using Dyneema® lines.

As illustrated in Fig. 15, the overall error behaviour remains comparable to that observed in the ceiling-suspended configuration, a summary of the results is shown in Table 10. For MoI values above $3\cdot 10^{-3}\;\mathrm{kg}\;m^{2}$, the error remains below 5%. For intermediate MoI values (10^−3^–$3\cdot 10^{-3}\;\mathrm{kg}\;m^{2}$), the error ranges between 5% and 15%. However, for MoI values below $10^{-3}\;\mathrm{kg}\;m^{2}$, the measurement error increases to approximately 40%. This behaviour is most likely caused by structural flexibility in the support frame, which introduces small additional oscillatory modes that become significant when measuring very small inertias (see Table 12).

To evaluate the implemented centre-of-mass (CoM) compensation function, additional measurements were performed using reference masses positioned at known lateral offsets from the platform centre. Tests were conducted using a single calibration mass (0.8088kg) displaced by 5 mm and 9 mm, and using two calibration masses (1.6176kg total mass) displaced by 15 mm and 20 mm. For each configuration, the MoI was calculated both with and without the CoM compensation enabled. The measured reduction in MoI produced by the compensation function was compared with the theoretical correction predicted by the parallel axis theorem ($md^{2}$). For all tested offsets, the applied correction closely matched the expected theoretical value. For example, the 9 mm offset case produced a measured correction of $6.56\times 10^{-5}\;\mathrm{kg}\;m^{2}$, compared with a theoretical value of $6.55\times 10^{-5}\;\mathrm{kg}\;m^{2}$. Similarly, the 15 mm and 20 mm offset tests produced corrections of $3.63\times 10^{-4}\;\mathrm{kg}\;m^{2}$ and $6.46\times 10^{-4}\;\mathrm{kg}\;m^{2}$ respectively, in close agreement with the corresponding theoretical values. Table 11 compares the correction applied by the software with the theoretical correction predicted by the parallel axis theorem. For all tested masses and offsets, the measured correction closely matches the expected value of $md^{2}$. The largest absolute difference between the measured and theoretical correction terms was less than $1\times 10^{-6}$ $\mathrm{kg}\;m^{2}$, confirming that the implemented centre-of-mass compensation algorithm correctly applies the parallel-axis correction and scales appropriately with both object mass and offset distance.

**Table 10:** Tests with the platform suspended from the purpose-built support structure using Dyneema® lines. Every test was repeated 5 times.

Theoretical MoI ${\mathrm{kg m}}^{2}$	Mean measured MoI ${\mathrm{kg m}}^{2}$	Std measured MoI ${\mathrm{kg m}}^{2}$	Percent error
0.0001547	0.0002194	2.54e−05	41.89
0.0010353	0.0011433	2.15e−05	10.44
0.0011772	0.001198	1.31e−05	1.77
0.0015415	0.0017395	2.92e−05	12.84
0.0020478	0.0022246	5.51e−05	8.64
0.0030853	0.0031458	3.21e−05	1.96
0.0042447	0.0041214	4.43e−05	2.9
0.004594	0.0047269	5.95e−05	2.89
0.0061028	0.0062197	3.69e−05	1.92

**Table 11:** Verification of the implemented centre-of-mass compensation algorithm.

Offset	Mass	Mean uncorrected	Mean corrected	Measured correction	Theoretical correction
mm	kg	${\mathrm{kg m}}^{2}$	${\mathrm{kg m}}^{2}$	${\mathrm{kg m}}^{2}$	${\mathrm{kg m}}^{2}$
5	0.8088	$9.296\times 10^{-4}$	$9.092\times 10^{-4}$	$2.04\times 10^{-5}$	$2.02\times 10^{-5}$
9	0.8088	$9.500\times 10^{-4}$	$8.844\times 10^{-4}$	$6.56\times 10^{-5}$	$6.55\times 10^{-5}$
15	1.6176	$3.041\times 10^{-3}$	$2.678\times 10^{-3}$	$3.63\times 10^{-4}$	$3.64\times 10^{-4}$
20	1.6176	$3.123\times 10^{-3}$	$2.477\times 10^{-3}$	$6.46\times 10^{-4}$	$6.47\times 10^{-4}$

### 7.2 Damping characteristics of the pendulum

**Fig. 16:**
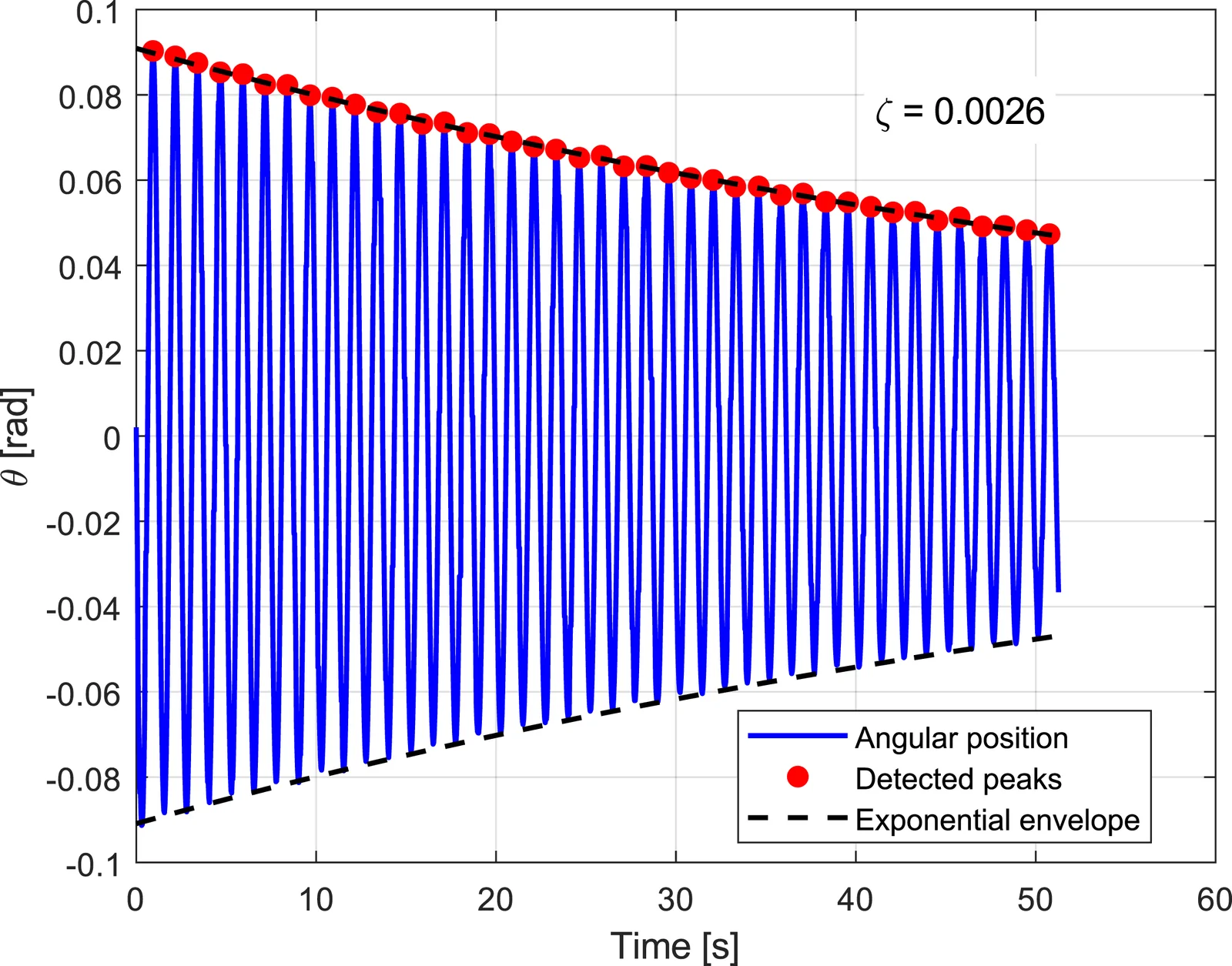
Free decay response of the Pendulum platform.

To assess the influence of damping on the oscillation period measurement, an additional free-decay experiment was conducted using the unloaded platform, shown in Fig. 16. The angular response was recorded for 50 s and analysed using a logarithmic decrement method. The measured logarithmic decrement was 0.0162, corresponding to a damping ratio of (ζ=0.00257), indicating a very lightly damped system. An exponential envelope was fitted to the peak amplitudes of the oscillation to characterise the decay behaviour. To determine whether damping introduced a measurable change in the oscillation period, the average period was calculated separately over the first and last 10 s of the recording. The measured period decreased from 1.2476 s to 1.2429 s, corresponding to a difference of only 0.0048 s (0.38%). Given that routine MoI measurements are typically based on recordings of approximately 20 s duration, these results indicate that damping has a negligible influence on the period estimation and, consequently, on the calculated moment of inertia. The observed damping is therefore not considered a significant source of measurement error for the proposed test setup. All validation measurements were performed indoors under stable laboratory conditions. The influence of ambient temperature, air currents, and day-to-day operator variability was not investigated separately and remains a topic for future work.

### 7.3 Validation with a representative CubeSat-like test article

**Fig. 17:**
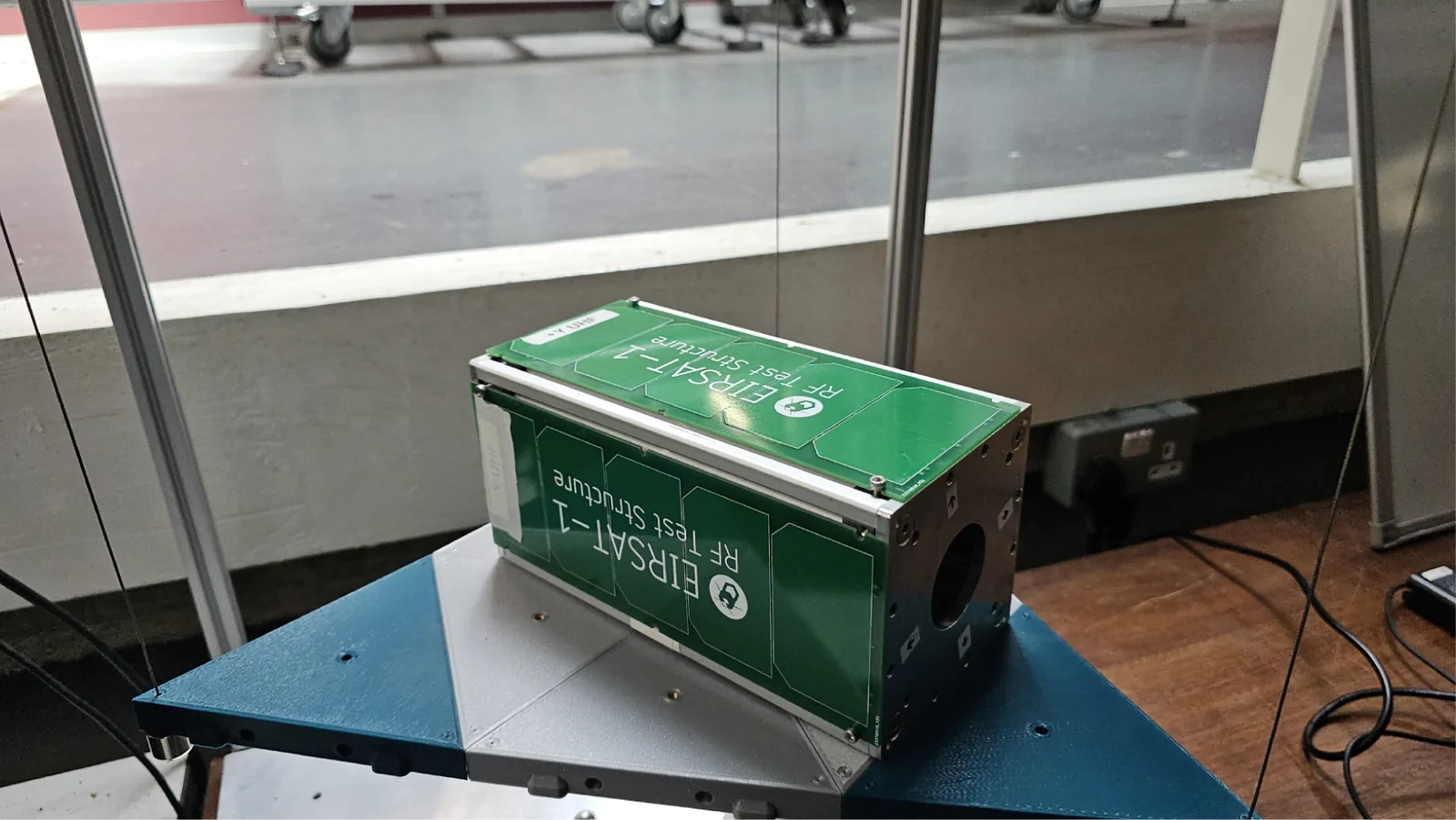
Representative CubeSat-like mock-up used to validate the proposed mass moment of inertia measurement system. The structure consists of an aluminium frame with PCB-based solar panel simulators and integrated calibration masses.

To evaluate the applicability of the proposed system to small satellite hardware, an additional validation test was performed using a representative CubeSat-like mock-up, shown in Fig. 17. The test article consisted of an aluminium structural frame with four PCB-based mock-up solar panels. Additional calibration masses were integrated into the structure to achieve a representative CubeSat-scale mass and inertia. A corresponding CAD model was generated and used to estimate the theoretical mass moment of inertia about the measurement axis. The measured MoI was found to be $6.738\times 10^{-3}\;\mathrm{kg}\;m^{2}$, while the CAD model predicted $6.996\times 10^{-3}\;\mathrm{kg}\;m^{2}$, corresponding to a difference of 3.7%. The observed discrepancy is attributed primarily to small assembly details that were not represented in the CAD model, including tape, fasteners, T-slot hardware, and minor assembly tolerances. Despite these simplifications, good agreement was obtained between the experimental and modelled results, demonstrating the suitability of the proposed system for the measurement of CubeSat-scale structures and assemblies.

**Table 12:** Recommended measurement configuration as a function of expected MoI range.

Expected MoI range	Recommended configuration	Notes
$I>3\times 10^{-3}$ kg m${}^{2}$	Support structure or ceiling suspension	Errors typically below 5%.
$1\times 10^{-3}\leq I\leq 3\times 10^{-3}$ kg m${}^{2}$	Ceiling suspension with Dyneema® lines	Reduces structural coupling; errors typically below 10%.
$I<1\times 10^{-3}$ kg m${}^{2}$	Modified setup recommended	Reduce platform inertia, increase suspension length, or use higher-resolution imaging.

### 7.4 Limitations of the trifilar pendulum model

The analytical formulation used throughout this work is based on the classical trifilar pendulum model and therefore relies on several simplifying assumptions. These assumptions are commonly adopted in trifilar pendulum analyses [5], [6] and were considered appropriate for the intended application; however, deviations from these assumptions may influence the measurement accuracy. First, the model assumes small-angle oscillations, allowing the motion to be approximated as linear. For large angular displacements, non-linear effects become increasingly significant and may alter the oscillation period [6]. To minimise these effects, all measurements presented in this work were performed using small initial angular displacements (<5°). Second, the suspension system is assumed to consist of three equal-length suspension lines arranged symmetrically about the platform centre. Differences in cable length, unequal tension distribution, or asymmetrical attachment locations may introduce coupling between rotational and translational modes of motion. Such effects can influence the measured oscillation period and contribute to increased measurement uncertainty. The model further assumes that the suspension lines possess negligible torsional stiffness and mass relative to the suspended system. Experimental results presented in Section 7 indicate that the choice of suspension material influences the measurement accuracy, suggesting that cable properties may contribute to deviations from ideal trifilar behaviour. The analytical formulation also assumes that the platform behaves as a rigid body. In practice, small structural deflections may occur within the platform, suspension attachments, or optional support structure. While these effects were not quantified through finite-element or modal analysis, they are believed to contribute to the increased measurement error observed for low-inertia test cases. Finally, accurate measurements require the centre of mass of the item under test to be located close to the geometric centre of the platform. Although a CoM offset correction based on the parallel axis theorem is implemented within the software, the accuracy of this correction depends on the accuracy with which the offset is known. Large offsets, unequal mass distributions, or significant translational sway may introduce additional uncertainty that is not explicitly captured by the current analytical model.

Although data processing is automated, measurement quality remains dependent on correct placement of the test article, appropriate alignment of the centre of mass with the platform centre, and excitation of the pendulum within the recommended angular range. The theoretical trifilar model assumes that the centre of mass of the item under test coincides with the centre of the platform. While the software includes an optional CoM correction based on the parallel-axis theorem, significant misalignment may increase uncertainty and should therefore be minimised. Previous studies by [5], [6] provide a more detailed treatment of CoM offsets and their influence on trifilar pendulum measurements.

These limitations are most significant when measuring objects with inertias approaching the lower measurement range of the system, where small deviations from the underlying assumptions represent a larger fraction of the measured quantity. Nevertheless, the validation results demonstrate that the system provides useful accuracy for the intended class of small satellites and similar rigid-body assemblies.

The printed paper fiducial markers may degrade through handling, contamination, or repeated removal and should be inspected or replaced when damaged.

### 7.5 Discussion

A variety of experimental approaches for inertia measurement have been reported in the literature, ranging from tetrahedron-based methods [8] and torsion pendulum systems [9] to more complex pendulum arrangements capable of determining the full inertia tensor [7], [10]. Compared with these systems, the proposed design prioritises portability, accessibility, and reproducibility through the use of additively manufactured components, camera-based optical tracking, and fully open-source hardware and software resources. While the system does not determine the complete inertia tensor, it provides a practical and low-cost method for measuring the principal mass moment of inertia of small satellites and similar rigid bodies.

The validation experiments demonstrate that the proposed system provides reliable MoI measurements for a wide range of small objects. For MoI values exceeding $3\cdot 10^{-3}\;\mathrm{kg}\;m^{2}$, the measurement error consistently remains below 5% regardless of camera type or suspension configuration. In the intermediate range between 10^−3^ and $3\cdot 10^{-3}\;\mathrm{kg}\;m^{2}$, measurement errors typically fall between 5% and 15%, which is acceptable for many engineering applications such as CubeSat inertia characterisation.

For very small inertias ($I<10^{-3}\;\mathrm{kg}\;m^{2}$), the measurement error increases significantly. In this regime, the inertia of the platform and suspension system becomes comparable to the inertia of the object under test, reducing measurement sensitivity. Additionally, small disturbances such as structural vibration, camera tracking resolution, and suspension dynamics contribute proportionally larger errors. The increased measurement error observed for low-inertia test cases is not attributed to a single source, but rather to the combined influence of several mechanical and measurement effects. First, as the MoI of the item under test decreases, it becomes small relative to the MoI of the platform. The measurement is therefore obtained from the difference between two larger quantities, namely the combined platform-plus-object inertia and the platform inertia alone. This reduces sensitivity and makes the final object MoI more susceptible to small errors in the measured oscillation period, mass, suspension geometry, and platform calibration.

Second, the suspension material influences the dynamic behaviour of the pendulum. The comparison between steel cables and Dyneema lines indicates that cable stiffness, mass, attachment method, and possible torsional effects can contribute to the measured error. These effects are expected to be most significant for low-inertia test cases, where small deviations from ideal trifilar-pendulum behaviour represent a larger fraction of the measured signal.

Finally, when the optional support structure is used, additional compliance may be introduced through the aluminium extrusion frame and its bolted bracket connections. The 20mm×20mm aluminium extrusion members provide a lightweight and portable frame; however, the T-slot joints and right-angle brackets are not perfectly rigid. Small structural deflections or low-amplitude frame vibrations may therefore couple into the platform motion. A detailed finite-element or modal analysis of the support structure was not performed as part of this work; consequently, the relative contribution of frame dynamics to the overall measurement error could not be quantified. Nevertheless, the observed increase in error for low-inertia measurements is consistent with the expected influence of structural compliance, suspension dynamics, and platform inertia.

The natural frequencies of the support structure were not measured in the present work. However, the observed increase in low-inertia measurement error when using the frame suggests that low-amplitude support-frame modes may couple into the pendulum motion. Future work will include experimental modal testing or finite-element analysis of the support frame to quantify this effect.

These observations suggest that the practical lower measurement limit of the system is governed by the combined effects of platform inertia, suspension dynamics, tracking resolution, and support-structure stiffness. For low-inertia objects, improved performance may be achieved by reducing the platform inertia, using suspension lines with low bending and torsional stiffness, increasing the rigidity of the support frame, or suspending the platform directly from a rigid ceiling structure. Future versions of the support frame could also include diagonal bracing members, larger extrusion profiles, or higher-stiffness joint connections to reduce parasitic structural motion while retaining portability.

An additional design consideration is the geometric ratio between the platform radius R and suspension length L. Previous investigations of trifilar pendulum dynamics [6], [13] have shown that smaller values of R/L reduce the influence of non-linear motion and improve agreement with the assumptions of the linear trifilar model. The experimental configuration employed an R/L ratio of approximately 0.18. Future implementations targeting lower inertia test articles could further reduce this ratio by increasing suspension length. In practical terms, this may be achieved by increasing the suspension length while maintaining the platform geometry. Although the present system was optimised for CubeSat-scale mass properties and a fixed suspension length was employed throughout testing. Further improvements to the lower measurement limit may also be achieved through reduction of platform inertia, optimisation of the suspension geometry (R/L ratio), and the use of higher frame-rate imaging systems. The relative contribution of these modifications should be quantified experimentally and through structural/modal analysis in future work.

### 7.6 Recommendations

Based on the experimental results, several practical recommendations can be made. For MoI values exceeding $3\cdot 10^{-3}\;\mathrm{kg}\;m^{2}$, the purpose-built support structure provides sufficient accuracy while offering practical advantages such as portability and ease of use.

For MoI values between 10^−3^ and $3\cdot 10^{-3}\;\mathrm{kg}\;m^{2}$, suspending the platform directly from a rigid ceiling structure using Dyneema® lines yields the lowest measurement error, typically below 10%. However, Dyneema lines may be susceptible to damage from sharp edges on printed components, which should be considered when testing valuable hardware.

For MoI values below $10^{-3}\;\mathrm{kg}\;m^{2}$, improved accuracy may require modifications to the experimental setup, such as reducing the platform inertia, increasing suspension length, or employing higher-resolution imaging systems. Consequently, increasing the suspension length may improve measurement sensitivity for lower-inertia test articles without requiring substantial modifications to the platform geometry. Alternative measurement methods may also be preferable for objects with extremely small inertias.

Future work will focus on improving the robustness and usability of the open-source system while retaining low-cost manufacturability. Potential improvements include automatic camera calibration and image rectification, more durable printed or engraved N-fold markers, a stiffer support structure incorporating diagonal bracing or larger extrusion profiles, and contamination control or low-outgassing materials. Future work could also include finite-element and experimental modal analysis of the support structure to quantify structural compliance, identify potential parasitic vibration modes, and evaluate their contribution to measurement uncertainty, and a repeatable release mechanism to reduce operator-induced sway. Additional work could also investigate environmental effects such as temperature variation, air currents, and long-term durability of Dyneema® suspension lines.

The proposed platform is intended as a laboratory measurement fixture for ground-based testing rather than as flight hardware. The PLA construction provides a low-cost and readily manufacturable solution suitable for most laboratory applications. When testing contamination-sensitive hardware, the platform should be visually inspected and cleaned in accordance with the applicable laboratory or cleanroom procedures before use. For applications requiring enhanced contamination control, the additively manufactured platform could alternatively be fabricated using engineering polymers with verified low-outgassing characteristics, such as Prusament® Space Grade Polycarbonate, or from conventionally machined materials, without altering the underlying measurement principle.

### 7.7 Conclusions

This paper presented a low-cost, open-source trifilar pendulum system for measuring the mass moment of inertia (MoI) of CubeSat-scale spacecraft and other small rigid bodies. The proposed system combines an additively manufactured platform, camera-based optical tracking using N-fold fiducial markers, and open-source analysis software to provide an accessible alternative to commercial inertia measurement systems. Validation was performed using calibration masses with analytically known inertia values across four experimental configurations, together with an additional CubeSat-like object representative of the intended application.

The experimental results demonstrate that the proposed system is capable of measuring MoI values greater than $3\times 10^{-3}$ kg m^2^ with errors below 5%, while typical errors between 5% and 15% were obtained for MoI values between $1\times 10^{-3}$ and $3\times 10^{-3}$ kg m^2^. For very small inertias ($<1\times 10^{-3}$ kg m^2^), measurement accuracy decreases due to the combined influence of platform inertia, suspension dynamics, and measurement sensitivity. Validation using a representative CubeSat-like test article showed a measured MoI within 3.7% of the corresponding CAD prediction, demonstrating the applicability of the proposed approach to spacecraft hardware. Together with the openly available design files, software, documentation, and validation data, the presented system provides a reproducible platform for experimental inertia measurements and aims to lower the barrier to accurate mass property characterisation within the small-satellite community.

## CRediT authorship contribution statement

**Bas Stijnen:** Writing – original draft, Software, Methodology, Conceptualization. **Joseph Thompson:** Writing – review & editing, Conceptualization. **Ryan Paetzold:** Writing – review & editing, Conceptualization. **Eoghan Somers:** Methodology, Conceptualization. **David McKeown:** Writing – review & editing, Supervision, Methodology.

## Declaration of Generative AI and AI-assisted technologies in the manuscript preparation process

During the preparation of this manuscript, the author(s) used ChatGPT and DeepSeek to assist with language refinement and clarity. The author(s) have reviewed and edited the output and take full responsibility for the content of this publication.

## Declaration of competing interest

The authors declare the following financial interests/personal relationships which may be considered as potential competing interests: Bas Stijnen reports financial support was provided by University College Dublin. If there are other authors, they declare that they have no known competing financial interests or personal relationships that could have appeared to influence the work reported in this paper.

## Appendix A Supplementary material

The supporting files are available for download on: https://data.mendeley.com/datasets/zww548rfbn/6.
